# *In vivo* two-photon microscopy for studies of neurovascular coupling in rodents: a beginner’s guide

**DOI:** 10.1117/1.NPh.12.S2.S22808

**Published:** 2025-11-04

**Authors:** Barbara Lykke Lind, Krzysztof Kucharz, Changsi Cai

**Affiliations:** University of Copenhagen, Department of Neuroscience, Faculty of Health and Medical Science, Copenhagen, Denmark

**Keywords:** Neurovascular coupling, two-photon imaging, awake mice, anesthetized mice, vascular signaling, endothelial cells, pericytes, vascular smooth muscle cells, astrocytes

## Abstract

Neurovascular coupling (NVC) ensures the precise delivery of blood to active brain regions and is vital for maintaining cerebral homeostasis. To investigate the dynamic complexity of NVC *in vivo*, two-photon microscopy (TPM) provides excellent spatial and temporal resolution, enabling detailed visualization of cell-specific interactions and signaling mechanisms in the intact rodent brain. This review details the application of TPM in vascular imaging. We describe surgical preparations and discuss methodological considerations crucial for differentiating vessel types and accurately capturing neurovascular dynamics. Furthermore, we discuss the integration of TPM with genetically encoded fluorescent indicators that promise further advances in elucidating NVC mechanisms in health and disease. Finally, we highlight the recent advances in cutting-edge imaging technologies, which are poised to drive future discoveries in cerebrovascular physiology and pathology.

## 1 Introduction

The dynamic regulation of cerebral blood flow (CBF) in response to neuronal activity is termed neurovascular coupling (NVC). It is widely believed to be critical for maintaining cerebral homeostasis, supplying blood to brain areas in response to increased metabolic demand.^1^^–^^3^ Disruptions in NVC are implicated in various neurological conditions, e.g., brain ischemia,^4^^,^^5^ and suggested to be a contributing factor in the progression of neurodegenerative diseases, e.g., Alzheimer’s.^6^^,^^7^ This connection underscores the necessity of comprehensively understanding its underlying mechanisms. Recent advances in imaging technologies, notably two-photon microscopy (TPM) in rodent brains, have substantially improved our insights into NVC processes *in vivo*. This review lists some important considerations for any investigator who wishes to conduct a vascular-related investigation using TPM.

TPM provides deeper tissue penetration and significantly reduced phototoxicity compared with conventional fluorescence imaging methods. This allows visualization of fine vascular structures at cellular and subcellular resolutions *in vivo* and longitudinal monitoring of vascular dynamics in the brain. Consequently, TPM has been extensively applied to investigate complex vascular responses, including dynamic vessel diameter changes, vessel connectivity, and vascular signaling with functional readouts, e.g., cellular ${\mathrm{Ca}}^{2+}$ dynamics and blood/brain tissue oxygenation,^8^^–^^10^ all of which are integral to NVC. Several comprehensive reviews have previously detailed the technical principles of TPM for *in vivo* studies,^11^^,^^12^ intricate signaling pathways involved in NVC,^1^^,^^2^^,^^13^ and classical TPM methodologies for investigating rodent vascular function.^14^^,^^15^

This review serves as an introductory guide, addressing practical and scientific considerations as well as optimized approaches for applying TPM to the study of NVC. We provide a critical description of different nomenclatures for cerebral vasculature and offer guidelines for identifying and classifying vascular segments using TPM. Following a concise introduction to key cell types and signaling mechanisms involved in NVC, we discuss tailored strategies for investigating each vascular cell type. Among the methodological considerations are the choice between chronic versus acute and awake versus anesthetized animal experimentation. The review concludes by highlighting emerging fluorescent indicators and imaging modalities, emphasizing the ongoing need for technological and methodological progress to further enhance vascular research and ultimately improve therapeutic strategies for cerebrovascular disorders.

## 2 Principles of Two-Photon Microscopy in Vascular Imaging

### 2.1 Advantages and Disadvantages of *In Vivo* Two-Photon Microscopy

TPM has emerged as a powerful tool for *in vivo* vascular imaging, offering several advantages over traditional confocal microscopy. It uniquely integrates several critical analytical capabilities, i.e., superior spatial information, subcellular resolution, temporal precision, longitudinal monitoring, and real-time data acquisition in the living, intact brain.^14^^,^^16^^–^^18^

The main advantage of TPM is its superior tissue penetration. In contrast to confocal microscopy, which is strongly limited by light scattering in brain tissue, TPM employs longer wavelengths (typically in the infrared range) that scatter less, thereby improving image quality at greater depths and producing higher signal-to-noise ratios. These benefits are particularly advantageous for studying vascular networks in the brain, where deep-tissue imaging is crucial.^19^^–^^22^ Indeed, in the rodent brain, imaging depths have been reported of 250  μm through a polished and reinforced thinned-skull window, 500  μm with dura-removed craniotomies at 800 nm excitation, and even 1 mm or more with longer excitation wavelengths (e.g., 1280 to 1300 nm).^19^^,^^21^^–^^23^ Another significant advantage is the reduced phototoxicity and photobleaching associated with TPM. Because excitation occurs primarily at the focal point, the surrounding tissue is exposed to significantly less photodamage and fluorophore degradation.^17^^,^^18^^,^^24^ This is critical for long-term *in vivo* studies, as it allows researchers to monitor vascular dynamics longitudinally, without compromising tissue integrity or fluorescent labeling.^25^^–^^27^ Finally, the highly localized two-photon excitation enables natural optical sectioning, allowing for the acquisition of images at multiple depths. This enables the generation of detailed 3D maps of vascular networks *in vivo* and is an approach frequently used for analyses of vascular architecture by branching patterns and connectivity.^15^

TPM is distinguished by its ability to deliver both high spatial and temporal resolution. Transmission electron microscopy (TEM) and immunohistochemistry provide exceptional spatial and subcellular resolution but lack temporal dynamics. Fiber optics and microdialysis yield strong temporal resolution, and in the latter, the ability to measure protein concentrations, but lack spatially resolved cellular or vessel-specific data. MRI and PET enable minimally invasive, longitudinal assessments but fall short in subcellular detail [discussed in Ref. 14]. Wide field hemodynamic methods, such as intrinsic optical imaging and laser speckle imaging, capture activity across wide brain areas but are restricted to superficial tissue volumes and lower spatial precision. Notably, TPM can be combined with these techniques, as optical access requirements are comparable, and such integration can add a valuable spatial perspective to neurovascular investigations.^28^^–^^30^

However, similar to every method, the TPM also has certain limitations. A primary disadvantage is the higher cost of instrumentation compared to traditional fluorescence imaging approaches, as multiphoton microscopes require specialized lasers and optics that are more expensive and complex to operate. Although TPM *in vivo* can achieve subcellular resolution, the spatial resolution with TPM can be slightly lower compared with confocal microscopy, particularly when longer excitation wavelengths are used for deeper penetration, as the diffraction limit scales with wavelength.^18^ This limitation is particularly noticeable when imaging fine vascular structures or subcellular details.^15^^,^^31^ The choice of fluorescent labels is another consideration, as not all fluorophores are efficiently excited by the two-photon laser, and some exhibit low quantum yields, resulting in weaker signals.^21^^,^^23^^,^^24^ The careful selection of appropriate fluorescent dyes or proteins is thus crucial for successful TPM imaging *in vivo*.

Effectively, the principal challenges of TPM require careful optimization of imaging parameters to avoid confounding artifacts. These include **(i)** laser-induced tissue heating and photodamage, **(ii)** immersion objective-associated temperature loss, and **(iii)** motion artifacts. To avoid **(i)**, the general guideline is to use the lowest possible laser power while maximizing detector sensitivity (PMTs) to maintain an adequate signal-to-noise ratio. Laser heating, closely linked to photodamage, can occur even without overt tissue injury. Although laser intensity is highest at the cortical surface, the deeper regions are also susceptible to damage, as the most pronounced heating arises several hundred micrometers deeper, as the surface tissue layers are cooled due to thermal diffusion through the cranial window.^32^ The photodamage of the fluorophores manifests as progressive photobleaching, whereas tissue damage is often revealed by increased background autofluorescence or, in more severe cases, by visible tissue lesions, particularly at the edges of the scanned field where dwell times of laser scanning are longest. Importantly, such damage can elicit immune responses that may persist beyond the immediate imaging session, even in the absence of overt autofluorescent signals. Previous work^33^ indicated that laser power under the objective at <50 to 60 mW is relatively well tolerated by the tissue when imaging is restricted to 10 to 15 min, although we emphasize that these parameters must be empirically established for each setup. **(ii)** The objective acts as a heat sink, cooling the exposed brain tissue under a craniotomy. Studies from Charpak’s and Water’s groups showed that without active heating, the exposed brain temperature may fall by ∼2°C to 3°C up to ∼10°C at room temperature,^34^^,^^35^ thereby altering neuronal activity, metabolism, CBF, and vessel diameter. Although this can be mitigated using an objective heater coupled to a feedback sensor,^35^ being aware of this constraint is important for performing stable and physiologically relevant measurements and interpretation of data of neural and vascular dynamics *in vivo*. Finally, **(iii)** motion artifacts represent a distinct limitation. Although increasing pixel dwell time or frame averaging improves signal-to-noise ratios, it introduces temporal blurring, thereby reducing the accuracy of measurements. This concerns not only fast-moving objects such as pulsatile blood vessels, but all entities, even in the brain parenchyma, because of the heartbeat-driven or behavior-associated movements of the entire brain tissue. As highlighted by Kutuzov et al., specific acquisition strategies can mitigate these limitations, enabling precise quantification of vessel diameters and structures superiorization, with accuracy surpassing the diffraction limit of the conventional TPM.^36^^–^^38^

Despite these limitations, TPM emerges as a robust and powerful imaging modality for *in vivo* studies, effectively combining critical spatial and temporal attributes essential for the comprehensive analysis of NVC.

### 2.2 Surgical Preparation of Rodents for Two-Photon Imaging

Most of the brain research *in vivo* is performed in rodents, typically in mice. Rats, compared with mice, have a larger brain size, which presents challenges in achieving significant imaging depths. Non-rodent models, such as zebrafish, offer alternative avenues^39^^–^^41^; however, for the purpose of maintaining methodological consistency, we will only discuss mice.

The primary requirement for performing a TPM-based NVC investigation is to establish optical access to the brain. This access can be achieved through various strategies, such as bone thinning, inserting a prism, a grin lens, or a cranial window.^42^ Most of these procedures require a recovery period between the surgical intervention and the imaging experiment. This is to ensure that the neurons, astrocytes, vascular mural cells, and endothelial cells retain their spontaneous and evoked intracellular $Ca^{2+}$ signaling,^29^^,^^43^^–^^46^ which are involved in NVC^47^^–^^49^ and highly sensitive to suboptimal microsurgical procedures.^42^^,^^50^ Importantly, when craniotomy is performed successfully, both cellular morphology and neurovascular function are preserved.

Successful preparation of an acute cranial window can provide stable platforms for functional TPM without inducing significant perturbations to cortical physiology. In an acute window, an approach used in terminal experiments, the glass coverslip may be placed to only partially cover the skull opening. The gap allows direct access to brain tissue for electrophysiological assessments or compound delivery via glass micropipette.^51^^,^^52^ If fully sealed with a glass coverslip to protect the brain and ensure long-term sterility of the preparation, a cranial window can be chronically implanted for longitudinal studies. Several studies have suggested that a silicone sheet can be used as an alternative to the cover glass,^53^ which facilitates tissue access in a chronically implanted window and may potentially enable pharmacological experiments to be performed over multiple days. However, this technique is challenging as the soft silicone is more difficult to handle and sterilize during the surgery than the glass, which increases the likelihood of contamination of the window. A more practical alternative is the insertion of an intracortical cannula, which permits topical pharmacological interventions across a chronically implanted window.^54^^–^^56^ Acute cranial windows can also be used in awake experiments, provided the animal undergoes surgical preparation and head-fixation training; animals are typically euthanized at the end of such experiments.^52^ In practice, most investigators select the simplest method that aligns with their experimental design. Acute experiments involving a single imaging session are usually performed under anesthesia with a partially covered cranial window, whereas repeated or awake imaging studies generally require a chronically implanted window.

**Fig. 1:**
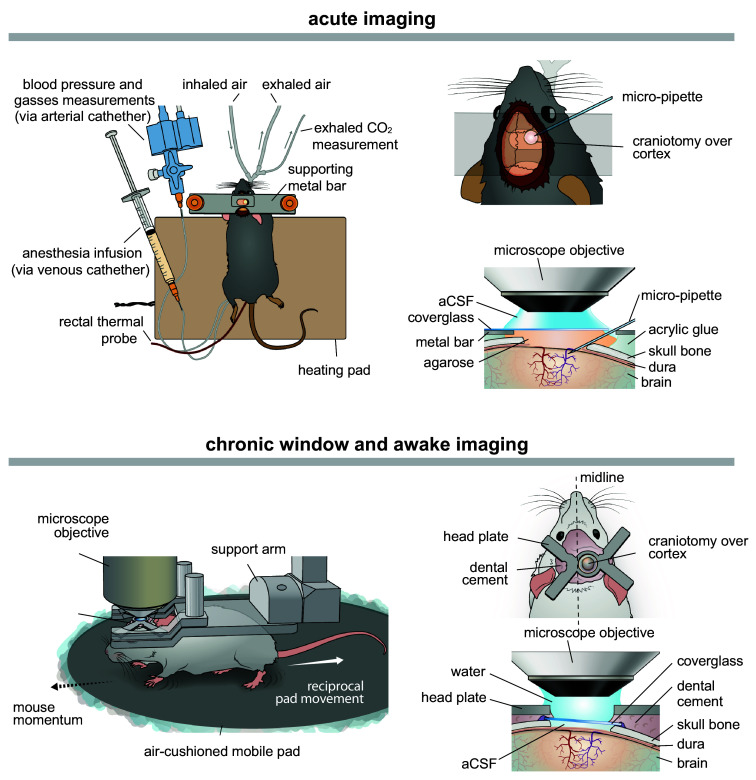
Diagram of microsurgical preparation and imaging platform for anesthetized and awake mice using TPM. Acute imaging (upper): The anesthetized mouse is maintained at constant body temperature by a heating pad, and the head is stabilized with a permanently attached headbar. Tracheotomy allows mechanical ventilation and continuous monitoring of exhaled ${\mathrm{CO}}_{2}$, whereas arterial and venous catheters permit blood pressure measurement, blood gas sampling, and infusion of anesthesia or other compounds, including fluorescent tracers. A cranial window with an opening enables insertion of a micro-pipette for electrophysiological recordings or application of vasoactive substances. Chronic window and awake imaging (lower): In chronic preparations, a fully sealed cranial window is implanted, and the skull is secured with a small head plate implant that enables repeated, detachable head fixation to the imaging platform. For behavioral support, animals are head-fixed with freely moving limbs on, e.g., an air-cushioned mobile platform, allowing recording of behavior in synchrony with two-photon imaging. For a detailed description, see Refs. 14, 30, and 51.

Besides the choice of optical access, the surgical procedure also differs substantially between experiments performed on awake animals and those conducted in terminal experiments under anesthesia (Fig. 1). To maintain physiological relevance in anesthetized preparations, additional surgical procedures are required to stabilize blood gases and pH throughout the experiment, to minimize unwanted systemic effects induced by anesthetics.^51^^,^^57^ Optimal blood parameters can be maintained by introducing mechanical ventilation, which supports the animal’s breathing while enabling continuous monitoring of expired ${\mathrm{CO}}_{2}$ to track metabolic changes and adjust levels as needed. This monitoring can be further supplemented with insertion of an arterial catheter, i.e., in the femoral artery, through which mean arterial blood pressure can be measured, and sampled for readouts of blood gas concentrations, e.g., oxygen, along with blood plasma pH.^51^ Finally, it is necessary to keep the body temperature physiological throughout the experiment, preferably by using a heating pad, autoregulated via feedback from a rectal probe (Fig. 1).

After completing the microsurgical preparations and confirming optimal physiological parameters, the animal must be head-fixed to ensure stable imaging. This entails attaching a headbar to the skull of the animal, which matches the holder on the imaging stage, keeping the head in a stable position below the objective. Various head-fixation systems have been developed; in our experience, those providing two or more points of attachment, anchoring both sides of the head, offer superior imaging stability and greater resistance to animal movement. For chronic and/or awake imaging, a small implant can be permanently affixed to the animal’s head, allowing secure but reversible fixation to the imaging platform. However, awake imaging requires an additional step of preparation: thorough training of the mice by the experimenters. Training is typically carried out over at least five days and progresses from simple handling to gradually increasing the duration of head fixation. The purpose is to habituate the mice to the noisy imaging environments and reduce anxiety, and accustom them to the stress of head fixation, as well as the specific behavioral platform used in the study. These behavioral platforms vary, ranging from a hammock or tube placed above unidirectional treadmills,^58^ which keep the animal awake but restrict natural movement,^52^ to more flexible multidirectional floating devices.^30^^,^^59^^,^^60^ In our experience, air-cushioned platforms (Fig. 1, lower panel) provide greater stability and comfort compared with setups in which the mouse must balance on a ball. These platforms can be expanded with a set of behavioral tasks, constituting the basis for more naturalistic experimental paradigms.^61^

## 3 Imaging the Brain Vasculature

### 3.1 Classification of Brain Vasculature

**Fig. 2:**
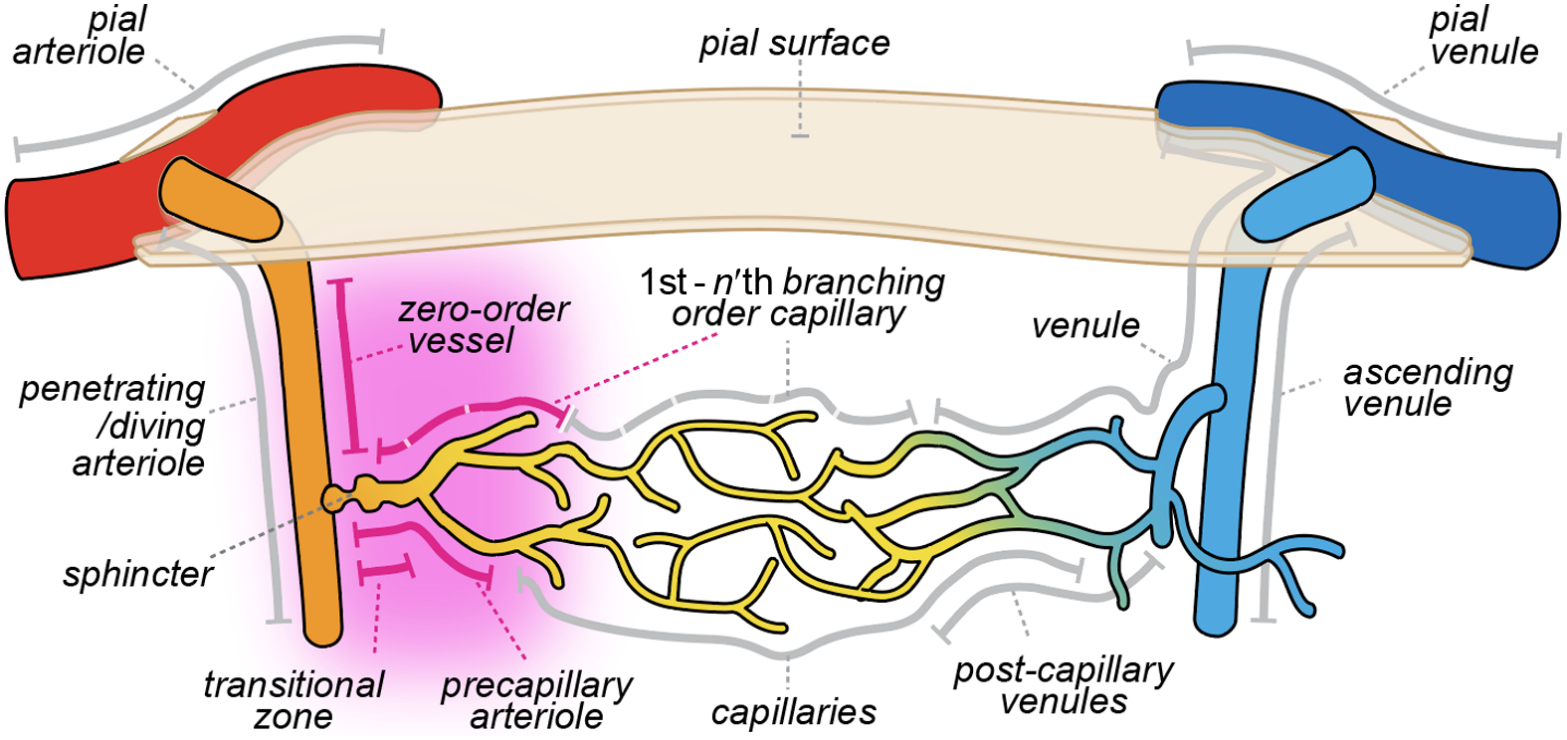
Distinct vessel types in the cerebral cortex, often described by conflicting and/or overlapping nomenclature. Gray lines indicate vessel segments and their corresponding names as reported in the literature, with overlapping lines denoting areas of overlap or inconsistency in terminology [see also Table 1]. The purple highlight demarks the most debated segment, variably referred to as the transitional zone, precapillary arteriole, or zero/first order capillary.

The rodent cerebral vasculature is a highly specialized network composed of distinct vessel types. Various nomenclatures have been proposed for vascular classification, complicating the comparison of vascular research studies. Accurate classification of vessels such as pial arterioles, penetrating arterioles, precapillary arterioles, capillaries of varying orders, and venules is complicated but vital for understanding NVC and blood flow regulation (Fig. 2).

The classification approaches can be grouped into four principal categories: morphology, molecular markers, function, and location. **(i)** Morphological classification relies primarily on vessel diameter, the presence or absence of specific structural features such as the internal elastic lamina, and the types of mural cells covering the vessel wall; **(ii)** Functional classification defines vessels based on their responses to stimuli, such as sensory stimulation, natural behavior, optogenetic activation, or pharmacological agents; and **(iii)** Molecular markers: using cell-type–specific promoters, fluorophores can be expressed in defined mural cell populations. Because these mural cells are restricted to particular vessel types, their visualization during imaging serves as a marker to identify the vessel type being examined. **(iv)** Location-based classification uses anatomical position in the vascular tree by counting the branching order and flow direction to classify the vessel.

However, each of these factors remains subject to separate debates, as morphological definitions may overlap, and distinct sets of molecular markers often yield conflicting interpretations of the same populations, as highlighted, e.g., for pericytes (as thoroughly discussed in Ref. 62). These challenges are not unique to imaging-based studies; even in non-imaging approaches such as spatial transcriptomics, vascular identity is frequently inferred from molecular markers that remain controversial. To maintain focus on TPM, we restricted our discussion to classification approaches directly derived from vessel imaging data. In our opinion, location-based classification is the most robust strategy for vessel classification in TPM studies. The approach requires a firm identification of the arterioles and penetrating arterioles. This can be based on morphological characteristics, but can also be judged from blood flow direction. The direction of the flow can be identified in a top-down approach, using wide-field inspection of the surface vessels within the craniotomy, then following the arterioles down, or a bottom-up approach, judging the flow direction by using a line scan of erythrocyte passages through smaller vessels,^63^ then counting capillary branch points backwards to the penetrating vessel. Ideally, vessel classification should integrate multiple complementary readouts, including flow direction, oxygenation (arterioles appear brighter than venules due to hemoglobin saturation when inspected through the craniotomy), and, where possible, selective cellular labeling. However, one needs to recognize that not all measurements are feasible simultaneously, and emphasis should be on providing a pragmatic and anatomically unambiguous identification of position in the vascular tree. Unlike the other approaches, it requires imaging in multiple focal planes and some skill in tracking the vessels, but it also provides a more assertive starting point for a neurovascular investigation. This approach is also more reliable than using only morphological cues such as vascular cell structure or vessel diameter, as these can be altered by age or pathology.

### 3.2 Variable Parameters Applied in the Classification of Brain Vasculature

These classification schemes, applied individually or in combination, provide a robust framework for dissecting microvascular structure and function. However, there is still a lack of consensus among different research groups regarding vessel nomenclature and classification. Below, we discuss commonalities, discrepancies, and ongoing debates in the field (Table 1). We suggest that, despite disagreement on nomenclature, authors should continue to provide accurate criteria for their terminology to allow comparison between studies.

**Table 1:** Summary of vessel classification criteria used in representative TPM and non-TPM vascular research studies frequently cited in the field. Although these studies also differ in additional parameters (e.g., animal preparation, imaging conditions, analysis strategies), these were not included, as they were not critical for vessel identification.

Source	Pial arterioles	Penetrating arterioles	Precapillary arterioles/transitional zone	Capillaries	Post-capillary venules	Ascending venules	Pial venules	Alternative terms/merged categories/other	Key methods/datasets	Classification criteria
Attwell et al.^64^	Mentioned in the context of the vascular bed.	Mentioned as branch order 0 preceding the capillary segment.	Term viewed as vague and confusing; preferring to omit it. “First capillary” (sometimes called precapillary arteriole) is designated as branch order 1.	Defined as vessels with spatially isolated contractile pericytes; branching order used, starting at 0 (penetrating arteriole) or 1 (first capillary branch).	Mentioned as a segment containing pericytes, but not explicitly classified	Not explicitly discussed/classified	Not discussed/classified	—	Various	Review/opinion letter
Blinder et al.^65^	Surface vessels traced to the middle cerebral artery; some were manually labeled as pial arterioles.	Vectorized as degree 2 to 3 nodes linked by centerlines; traced to the middle cerebral artery.	Mentions primary microvessels branching from penetrating vessels	No specific criteria for classification are detailed beyond being part of the vectorized network connecting penetrating vessels.	Not explicitly defined as a separate vessel type with specific criteria	Distinguished from arterioles by tracing connections to the central sinus or rhinal vein.	Identified as surface vessels. Distinguished from arterioles by tracing connections to the central sinus or rhinal vein.	Communities (topological grouping)	*Ex Vivo* imaging	Location, connectivity, morphology, topology, flow
Bonney et al.^66^	Penetrating vessel origin at pial surface noted in one dataset; lacks detailed classification.	Vessel wall includes endothelium, smooth muscle, fibroblasts; origin at pial surface visible.	Arteriole-capillary transition from penetrating arteriole to capillary bed; contains endothelium, sphincter/ensheathing pericytes, and fibroblasts.	Intervening vessels include endothelium, pericytes, fibroblasts, show junctions, caveolae, and basement membrane.	Thinner than arterioles, with endothelium, mural cells, fibroblasts; includes deep venules like principal cortical venules.	Described with post-capillary venules; shares wall composition—endothelium, mural cells, fibroblasts.	Not explicitly classified with detailed criteria within EM data.	Microvascular zones (arterioles, capillaries, venules, transitional segments)	MICrONS EM dataset	Location, morphology, ultrastructure, associated cells, subcellular details, non-cellular structures
Cai et al.^47^	Present, but not explicitly classified.	Identified by tracing to pial arterioles; show continuous smooth muscle rings; diameter 15.09±4.15 μm, larger than capillaries.	Not explicitly defined as a separate zone. The source focuses on capillaries branching off the penetrating arterioles.	Microvessels branch from penetrating arterioles: 1st order (7.18±1.93 μm), 2nd (6.25±2.43 μm), 3rd (7.63±2.47 μm); order starts at 1.	Not discussed/classified	Not discussed/classified	Not discussed/classified	—	TPM	Location, branching/connectivity; associated cells
Cai et al.^67^	Not discussed/classified.	The parent vessel from which capillaries branch (PA). Diameters for PA are mentioned alongside capillary orders ∼14 μm.	Not a separate zone; sphincter diameter (∼11.6 μm) compared to PA and capillaries, but the definition is not detailed.	Classified by branching: 1st order from penetrating arteriole; diameters ∼7.2 μm (1st), ∼6.3 μm (2nd), ∼5.8 μm (3rd).	Not discussed/classified	Not discussed/classified	Not discussed/classified	—	TPM	Connectivity/branching pattern, diameter
Chen et al.^68^	Identified as MCA/ACA branches on the cortical surface; perfusion dynamics mapped.	Mentioned as branches from MCA, monitored for perfusion drop; not separately defined.	Not explicitly classified.	Not individually resolved (resolution ∼14 μm); perfusion changes inferred at network level.	Not discussed/classified	Not discussed/classified	Surface venous drainage visible in perfusion maps, not systematically classified.	Core/penumbra mapping; collateral recruitment emphasized.	LHMI, LSCI, ex vivo staining	Location (arterial vs venous perfusion); perfusion thresholds (bfi <40% = core, 40–60% = penumbra); collateral recruitment.
Fernandez-Klett et al.^69^	Classified as arterioles - segments populated by Smooth Muscle Cells (SMCs).	Classified as arterioles - segments populated by Smooth Muscle Cells (SMCs).	Not a distinct zone; analyzes diameter to distinguish SMC-covered arterioles from pericyte-covered capillaries, including between pericyte bodies.	Segments with pericyte bodies or between them; classification distinguishes arterioles (SMCs) from capillaries (pericytes). Branching order (0 or 1) not addressed.	Not discussed/classified	Not discussed/classified	Not discussed/classified	—	TPM	Associated cells, functional
Gould et al.^70^	Distinguished by a diameter >10 μm and a location <100 μm from the surface.	Labeled as “penetrating” or “descending” arterioles; traced from surface arterioles to capillary bed. Defined by location, <3 branch orders from large arteriole, and >6 μm diameter.	Not distinguished from post-arteriole capillaries; 6 μm cutoff used. Precapillary zones may be included in <3 branch order PA definition; smooth muscle vs pericyte labeling needed.	Labeled as capillaries if <6 μm and traced from arterioles. Separated from PAs by branch order and diameter; pre-/post-capillary not distinguished. Branch order used within the capillary bed is not detailed.	Not morphologically distinguished from penetrating venules; required molecular markers. Reverse size cutoff applied from the venous side.	Labeled, separated by venous-side branching and diameter: <6 μm for capillary–venule, <12 μm at <100 μm depth for venule–pial vein.	Labeled, distinguished from penetrating venules by <12 μm diameter at 100 μm depth and surface location. Segregated by size and venous branching.	—	TPM/ex vivo/modeling	Location, connectivity/branching pattern, diameter
Grant et al.^71^	SMC-covered vessels treated as arterioles; diameter: 16.2±1.3 μm	SMC-covered vessels treated as arterioles; diameter: 16.2±1.3 μm	Not a distinct zone; mural cell types: ensheathing, mesh, thin-stranded pericytes (EP, MP, TSP) span arteriole-capillary-venule continuum. EPs on larger capillaries; diameter: 9.0±0.4 μm.	Classified by pericyte type: EP (∼9.0 μm), MP (∼6.3 μm), TSP (∼4.9 μm). Pericyte traits vary along capillary bed; continuum from SMCs to mid-capillary pericytes. Branching order not defined but linked to cell-type shifts.	Not explicitly classified; mural cell continuum extends into venules.	Not explicitly classified.	Mural subtypes—SMC, EP, MP, TSP—associate with vessel diameter and position; morphology and coverage form a continuum.	—	TPM/ex vivo imaging	Mural cell subtypes
Glück et al.^72^	Identified by diameter (100 to 200 μm high-order branches, 50 to 100 μm smaller arterioles); ACA/MCA/PCA inflow resolved.	Not explicitly imaged; pia-level network emphasized	Not discussed/classified	Only partially resolved; <5 μm capillaries not reliably measured.	Quantified as venous outflow pathways; diameters and flow directions mapped.	Not explicitly separated.	Surface veins distinguished from arteries by flow direction and slower velocity.	“Pial vascular connectome”; collaterals as functional units.	Pia-FLOW	Defined by vessel diameter, flow velocity, and flow direction
Grubb et al.^43^	Distinctions from pial arterioles are not detailed here.	Classified as vessels before sphincter/1st capillary branch; α-SMA positive; ∼14 μm diameter.	Identified as precapillary sphincter at 1st capillary branch from a penetrating arteriole; ∼11.6 μm with local narrowing. Distinct “Bulb” (∼15.2 μm) at same branch point.	Classified as capillaries branching from penetrating arteriole; focuses on 1st order capillary (∼7.2 μm) from sphincter/Bulb. Branching starts at 1. FITC-lectin positive (lumen), NG2 positive (pericytes)	Not discussed/classified	Not discussed/classified	Not discussed/classified	—	TPM	Location, connectivity/branching pattern, associated cells/morphology, histology, functional
Hall et al.^48^	Location-based identification	Identified in vivo by tracing to pial arterioles with smooth muscle and RBC flow into capillaries; designated 0th order.	Not a distinct zone; capillaries are classified by branching from penetrating arteriole—0th (penetrating arteriole) to 1st order (capillary).	Classified by branching: 1st order from 0th (PA), then 2nd, etc. Diameter changes and responses studied in 0th–2nd order vessels. Capillaries with/without pericytes compared; dilate faster than arterioles.	Not discussed/classified	Not discussed/classified	Not explicitly classified.	—	TPM	Location, connectivity/branching pattern, associated cells, flow, functional
Hartmann et al.^73^	Not explicitly classified with specific criteria.	Not explicitly classified with specific criteria.	Discussed as part of recent vascular categorization; includes “transitional zone” with distinct mural cells.	Focuses on capillaries; measures diameter, RBC velocity/flux. Notes small versus large capillaries and transit time heterogeneity. Branching order not used; shows diameter variability.	Not explicitly classified.	Not explicitly classified.	Not explicitly classified.	4-zone model: arterioles, transitional zone, capillaries, venules.	TPM	Functional, morphology, associated cells
Hartmann et al.^74^	Discussed as part of the 4-zone model as “arterioles”; contains distinct mural cells (SMCs).	Discussed in the context of the 4-zone model as arterioles, which contain distinct SMCs.	Explicitly defined as “transitional zone” in the 4-zone model; between arterioles and capillaries, with distinct mural cells.	Discussed as part of the vascular network, corresponding to the “capillaries” category in a simplified 4-zone classification. Contains distinct mural cells (pericytes).	Discussed as “venules” in the 4-zone model; contains distinct mural cells.	Discussed as “venules” in the 4-zone model; contains distinct mural cells.	Part of the 4-zone model: arterioles, transitional zone, capillaries, venules; argued to be overly simplistic.	—	Various	Review/opinion letter
Hatakeyama et al.^75^	Studied as a location for vasodilation; compared the cortical surface (pial) versus parenchyma.	Studied as a location for vasodilation; compared the cortical surface (pial) versus parenchyma.	Not explicitly defined as a separate zone or vessel type.	Not discussed or classified as a separate vessel type, but parenchyma measurements implicitly involve capillaries and other microvessels.	Not discussed/classified	Not discussed/classified	Not explicitly classified.	—	TPM	Location, cortical surface versus parenchyma, functional
Hill et al.^76^	Not explicitly classified with specific criteria.	Not explicitly classified with specific criteria.	Not a defined zone; the transition is inferred from the location of terminal SMA+ cells based on branch order and distance from the branch point.	Classified by vessel branch order; terminal SMA+ cell distribution and percentage per order analyzed. Start of branch order (0 or 1) not specified but likely corresponds to the capillary tree origin from the arteriole.	Not discussed/classified	Not discussed/classified	Not discussed/classified	—	TPM/ex vivo imaging	Connectivity/branching pattern, diameter, associated cells/molecular.
Iliff et al.^77^	Vessels studied via tracer distribution from the subarachnoid space; paravascular accumulation noted. No explicit classification given.	Vessels analyzed for tracer spread from the subarachnoid space; paravascular buildup noted. No specific type classification provided.	Not explicitly defined as a separate zone or vessel type.	No type classification or branching order described.	Vessels examined for tracer spread from the subarachnoid space; paravascular accumulation noted. No specific type classification.	Tracer paravascular accumulation noted. No specific type classification.	Vessels studied for subarachnoid tracer spread; paravascular accumulation noted. Types not explicitly classified.	—	TPM	Location, perivascular space
Mathiesen-Janiurek et al.^78^	Classified based on morphology and location, no specific criteria provided.	Classified based on morphology and location, blood flow direction, no specific criteria provided.	Not explicitly defined as a separate zone or vessel type.	Classified based on morphology and location. Specific criteria details or use of branching order (starting from 0 or 1) are not specified.	Classified based on morphology and location, no specific criteria provided.	Classified based on morphology, location, and blood flow direction, no specific criteria provided.	Classified based on morphology and location, no specific criteria provided.	—	TPM	Morphology, location, flow
Meng et al.^79^	Identified as surface arterioles; flow velocity measured up to ∼49 mm/s. Not explicitly classified with specific criteria.	RBC flux and velocity recorded; vessels tracked to >800 μm depth. Not explicitly classified with specific criteria.	Not explicitly defined as a separate zone or vessel type.	Classified by diameter (3 to 10 μm); RBC flux/velocity measured; cortical layer-dependent differences (superficial, intermediate, deep).	Identified by lower pulsatility compared to arterioles; velocity measured.	Not explicitly labeled, venular outflow included.	Surface venules imaged and compared to arterioles; slower flow.	RBC pulsatility indices; velocity profiles;	FACED TPM,	Morphology, vessel diameter, depth, flow direction, pulsatility
Kucharz et al.^31^	Classified as pial arterioles based on second harmonics generation, location, size	Classified as penetrating arterioles based on tracing from pial arterioles	Not defined as a separate zone, but capillaries from precapillary arterioles (≤10 μm) are noted. Supplementary info discusses capillary branching orders.	Diameters range 5 to 10 μm; ∼6th order branches noted. Branching starts not specified, but 1st-order from precapillary arterioles implied. Terminal (∼6th) branching orders mentioned for capillaries	Vessels with >6 μm diameter, downstream converging microvessels without capillary loops.	Coalescing from post-capillary venules, oriented perpendicular to brain surface, blood flow direction	Large vessels on the brain surface, slower blood flow than arterioles, lack of single harmonics generation	—	TPM	Location, morphology/diameter, connectivity/branching, functional, fluorescence/second-harmonic
Kucharz et al.^80^	Classified as a part of Blood-CSF Barrier vessels, based on location, morphology, branching, diameter, flow; diameter measure	Classified as a part of Blood-CSF Barrier vessels, based on location, morphology, branching, diameter, flow direction; diameter measure	Not explicitly defined as a separate zone or vessel type.	Classified based on morphology and location. Specific criteria details or use of branching order (starting from 0 or 1) are not specified. Considered as the BBB location	Grouped with ascending venules for mapping.	Grouped with post-capillary venules for mapping.	Classified as a part of Blood-CSF Barrier vessels, based on location, morphology, branching, diameter, flow direction; diameter measure	Cortex divided into superficial (0−60 μm), intermediate (60 to 120 μm), and capillary bed (120 to 180 μm) zones.	TPM/ex vivo imaging	Location, morphology, connectivity/branching pattern, diameter, flow, fluorescence/second-harmonic
Lacoste et al.^81^	Not explicitly classified with specific criteria.	Not explicitly classified with specific criteria.	Not explicitly defined as a separate zone or vessel type.	Classified as Layer IV vasculature/capillaries; 3D vessel density, branching, and diameter analyzed. Branching order not detailed as classification; diameter measured without specific cutoffs.	Not discussed/classified	Not discussed/classified	Not discussed/classified	—	Ex vivo imaging	Location, morphology/diameter, connectivity/branching, molecular
Lind et al.^82^	Classified as a penetrating arteriole (PA); studies the dilation dynamics relative to sphincter and capillary locations.	Classified as a penetrating arteriole; studies dilation initiation and spread to/from this segment, its location defined relative to the sphincter and capillary.	Explicitly identified as the “sphincter,” located between the penetrating arteriole and the first-order capillary.	Classified as capillaries, located downstream of the sphincter; termed the capillary compartment.	Not discussed/classified	Not discussed/classified	Not discussed/classified	Capillary compartment	TPM	Location:. Functional:. Fluorescence signal:
Longden et al.^9^	Identified as a capillary parent vessel and imaged in 4D; not explicitly classified beyond being larger and upstream of capillaries.	Identified as a capillary parent vessel and imaged in 4D; not explicitly classified beyond being larger and upstream of capillaries.	Not a distinct zone. Transition from penetrating arteriole to 1st order capillary implicit. Average inter-branch length (∼35.6 μm) matches endothelial cell length (∼34.9 μm).	Branching order from 1st to 5th (0th = PA). Quantifies branch point density ($∼4924/mm^{3}$), inter-branch length (∼35.6 μm), and EC nucleus spacing (∼34.9 μm), mapping Ca2+ events and RBC flux/velocity.	Not discussed/classified	Not discussed/classified	Not discussed/classified	Capillary endothelial cells and branch distinction with proto, unitary, and compound Ca2+ events.	TPM	Location, connectivity/branching pattern; morphology/topology; functional, fluorescence signal
Mughal et al.^83^	Included in the brain vasculature schematic; smooth muscle and endothelial cells labeled.	Included in a schematic overview of brain vasculature organization. Referred to as parenchyma arterioles.	Detailed discussion defines the post-arteriolar transition zone as a functionally distinct capillary region regulating blood flow, highlighting misuse of “precapillary arteriole” term.	Discusses the 1st-order capillary entering the bed splitting into two 2nd-order branches, which further branch into 3rd-order branches, continuing this pattern before converging on venules. The shortest path from penetrating arteriole to venule averages ∼7 branches.	Included in a schematic overview, but not explicitly defined.	Included in a schematic overview, but not explicitly defined, connects to capillary network.	Not explicitly discussed.	Feeding arteriole, draining venule, with additional focus on the retina.	Various	Review/opinion letter
Nishimura et al.^84^	Not explicitly classified; implicitly included as vessels upstream, parallel, or unconnected to a target penetrating arteriole in flow redistribution studies.	Studied as occluded “target penetrating arteriole”; other microvessel flow categorized by connectivity to it; visually identified as spiraling vessel entering parenchyma.	Not explicitly defined as a separate zone, but downstream vessels grouped by branch number (D1–D10), reflecting flow impact and likely including transitional zone and capillary bed.	Classified as microvessels linked to target penetrating arteriole; downstream branches grouped by number from PA (D1–D10), reflecting topological distance. Branching order starts at 1 for D1. “Deep microvessels”	Not explicitly classified; implicitly includes vessels further downstream and unconnected to the target penetrating arteriole.	Not explicitly classified; implicitly includes vessels further downstream and unconnected to the target penetrating arteriole.	Not explicitly classified.	Deep microvessels	TPM	Connectivity/topology, functional, flow direction
Pfau et al.^85^	Not explicitly classified.	Not explicitly classified.	Not explicitly defined as a separate zone or vessel type.	Classified as capillaries; compared cortex versus median eminence. Morphology via CD31 staining; ERG+ endothelial nuclei and pericyte coverage quantified. Diameter measured (∼4 to 8 μm in cortex). Branching order not detailed.	Not discussed/classified	Not discussed/classified	Not discussed/classified	—	*Ex vivo* imaging	Location, morphology, diameter, associated cells/molecular
Sakadzic et al.^86^	Identified by straighter morphology, gradual branching. Confirmed via TPM tracing at pial surface into cortex; manually labeled.	Manually labeled tracing from pial surface into cortex; precede capillaries; classified as arterioles called “diving arterioles.”	Not explicitly defined, but capillaries start one or two segments away from the penetrating (diving) arterioles based on morphology.	Labeled as capillaries, located 1–2 segments from diving arterioles by smaller diameter and tortuosity. Branching order not primary; position relative to diving arterioles used. Smaller diameter than arterioles/venules.	Not explicitly classified; grouped with ascending venules	Labeled and classified as venules; manually traced from cortical depth to pial surface. Located downstream of capillaries with diameters larger than capillaries.	Identified by curvier, thicker morphology with sprouting vessels and traced from 2-photon data of surface vasculature.	Merged categories: arterioles (including penetrating), venules (including penetrating and ascending), and capillaries with microvascular segments between bifurcations.	TPM	Location, morphology, diameter, connectivity, flow/functional, fluorescence signal
Shaw et al.^87^	Mentioned as vessels with a cortical surface location.	Discussed as penetrating/diving arterioles based on anatomy; diameter (15 to 40 μm), depth, branch order (0 from pial surface). Contain elastic lamina, elastin, SMC layers.	Discussed as precapillary sphincters, precapillaries, or transitional/low branch order capillaries—part of a continuum with gradual transitions.	Classified as capillaries with gradual anatomical changes. Diameter, depth, and branching order measured; branch order starts at 1 from penetrating arteriole and increases with bifurcations (C1–C4+). Includes pre-, mid-, and post-capillaries with distinct pericytes.	Discussed as vessels with gradual transitions; located after the capillary bed, leading to venules; associated with distinct mural cells.	Classified as vessels coalescing from post-capillary venules with gradual transitions, >50 μm)	Not explicitly defined	Pre-, transitional, low branch order, mid-, post-capillaries.	Review/Opinion Letter	Location, depth, morphology/microanatomy, connectivity/branching pattern, associated cells, functional, fluorescence signal
Shih et al.^15^	Reviewed TPM imaging methods for blood flow dynamics; no specific classification criteria detailed.	Reviewed TPM imaging methods for blood flow dynamics; no specific classification criteria detailed.	Not discussed/classified	Reviewed TPM imaging methods for blood flow in single vessels and capillary networks; includes flow parameter calculations. No specific classification criteria or branching order described.	Not discussed or classified as a separate vessel type with specific criteria.	Not discussed or classified as a separate vessel type with specific criteria.	Reviewed TPLSM methods for studying blood flow dynamics in cortical surface microvessels; no specific classification criteria provided.	—	TPM focus	Review/opinion letter
Watson et al.^88^	Not explicitly classified; focus on precapillary arterioles and capillaries.	Implicitly classified as a vessel giving rise to precapillary arterioles; indicated in the diagram but not separately defined.	Classified as a precapillary arteriole, compared with capillaries. Located along a precapillary arteriole near a penetrating arteriole. Mean diameter ∼10 to 12 μm. Branch order used for binary precapillary vs capillary classification.	Classified as capillaries, compared with precapillary arterioles. Mean diameter ∼5 to 6 μm. Branch order used statistically for binary pre-capillary versus capillary classification; detailed branching hierarchy not described.	Not discussed/classified	Not discussed/classified	Not discussed/classified	—	TPM	Morphology/diameter, connectivity/branching pattern, fluorescence signal, associated cells

#### 3.2.1 Pial arterioles

The pial arterioles spread across the brain’s surface, acting as the primary feeders for the vascular tree. Their location on the pial surface is the most common identifier, but here, they have to be distinguished from pial venules. As mentioned above, the color reflecting oxygenation (wide-field imaging, not TPM imaging) or the flow direction can be used. Connectivity is another key criterion; e.g., Blinder et al. trace them back to the middle cerebral artery. Morphology provides a tentative criterion, with Sakadzic et al. noting that they have a straighter appearance compared to venules.^65^^,^^70^^,^^86^^,^^87^ Diameter is frequently used, but thresholds vary considerably: Gould et al. use >10  μm, whereas Grant et al. measure an average of ∼16.2  μm for smooth muscle cell (SMC)-covered vessels (which include pial arterioles), and Shaw et al. discuss a range of 15 to 40  μm.^70^^,^^71^^,^^87^ This wide range reflects a significant difference in classification approaches. The presence of SMCs is a shared understanding,^71^^,^^87^ often described as continuous rings.^43^^,^^47^^,^^66^^,^^71^ Some studies utilize indirect imaging techniques, e.g., Kucharz et al., monitor second-harmonic generation (SHG) for pial arteriole identification.^31^^,^^45^ However, many studies do not provide explicit classification criteria for pial arterioles, often focusing on the deeper penetrating vessels.

#### 3.2.2 Penetrating arterioles

Diving from the surface into the cortex, these vessels, sometimes called “diving arterioles,”^86^ are critical for delivering blood deep below the pial surface. A common identification method is tracing their origin from the pial arterioles.^31^^,^^46^^–^^48^ Their defining feature is the presence of SMCs. However, diameter measurements show discrepancies: Gould et al. set a low bar at >6  μm, whereas Cai et al. find ∼15  μm, Grubb et al., ∼14  μm, Grant et al. ∼16.2  μm, and Shaw et al. use a broad 15 to 40  μm range.^43^^,^^47^^,^^71^^,^^87^ Branching order definitions also differ: Hall et al. designated the entire penetrating arteriole as the 0th order. By contrast, Gould et al. classify penetrating arterioles as vessels extending up to three branch orders from a large arteriole, whereas Shaw et al. employ a system that denotes branching along the penetrating arteriole itself.^43^^,^^48^^,^^70^^,^^82^^,^^87^

#### 3.2.3 Precapillary arterioles/transitional zone

This segment, bridging the SMC-wrapped arterioles and the pericyte-ensheathed capillaries, is perhaps the most controversially defined part of the network. Some researchers, similar to Attwell et al., view the term “precapillary arteriole” as vague and prefer to avoid it.^64^ Many other studies lack an explicit definition of this zone. Yet, a growing body of work identifies a distinct transitional region. Some focus on specific structures: Grubb et al., Cai et al., and Lind et al. identify a “precapillary sphincter” (∼11.6  μm) at the initial segment of the first-order capillary branching from an arteriole, a structure also noted by Bonney et al.^43^^,^^66^^,^^67^^,^^82^ Others define a broader “transitional zone,”^74^^,^^87^ often characterized either by large capillaries (∼9.0  μm) branching from arterioles,^71^ or simply by the vascular segment where morphologies of mural cells transit from a ring-shaped structure with flat soma to an ensheathing shape with ovoid soma^69^ (A detailed classification of vascular mural cells is provided in the following section). Watson et al. classify “precapillary arterioles” based on a diameter of ∼10 to 12  μm.^88^ The core debate centers on whether this is a discrete, definable zone or merely a gradual continuum, as emphasized by Shaw et al. and Grant et al.^71^^,^^87^

#### 3.2.4 Capillaries

Constituting the vast exchange network, capillaries are primarily defined by the presence of pericytes rather than a continuous SMC layer.^64^^,^^66^^,^^69^^,^^71^ Branching order is a very common classification method,^9^^,^^47^^,^^48^^,^^67^^,^^87^ but definitions vary regarding the starting point (0 or 1) and the number of branches considered. Diameter is also widely used, but cutoffs are inconsistent across studies: Gould et al. use 6  μm, whereas Watson et al. used ∼5 to 6  μm, Kucharz et al. used 5−10  μm, and Cai et al. provided ranges around 5.8 to 7.6  μm depending on branch order.^31^^,^^47^^,^^67^^,^^70^^,^^88^ Grant et al. offered a unique classification based solely on pericyte subtypes, i.e., ensheathing, mesh, thin-stranded pericytes, with associated diameters ranging from ∼4.9 to ∼9.0  μm.^43^^,^^71^ Morphology, such as increased tortuosity, can also be a factor.^86^ Notably, despite being the focus of many studies, several lack specific classification criteria beyond network inclusion.

#### 3.2.5 Post-capillary venules, ascending venules, and pial venules

The venous side of the microcirculation generally receives less detailed classification than the arterial side, and definitions are often imprecise or absent. Post-capillary venules mark the start of drainage. They are described as having thinner walls than arterioles but similar cellular composition.^66^ Kucharz et al. used a >6-μm diameter and converging flow.^31^ However, many studies do not distinguish them explicitly or, such as Gould et al., rely on reverse tracing with diameter cutoffs (6  μm) and note the need for molecular markers.^70^ Ascending venules collect blood and move it towards the surface. Their orientation and flow direction are key identifiers.^31^^,^^86^ Blinder et al. traced them to major sinuses. They are frequently grouped with post-capillary venules,^65^^,^^86^^,^^89^^,^^90^ blurring their distinct classification. Pial venules are the surface collectors. Similar to pial arterioles, their surface location is key.^31^^,^^65^^,^^70^^,^^89^ Blinder et al. used connectivity to the sinuses. Sakadzic et al. noted their curvier, thicker morphology compared with pial arterioles. Gould et al. used a cutoff diameter of greater than 12  μm, whereas Kucharz et al. employed diameter, slower flow, and second-harmonic imaging signal.^31^^,^^45^^,^^65^^,^^70^^,^^86^^,^^90^

### 3.3 Recommendation for the Identification of Brain Vasculature in NVC Studies

As described above, several classification approaches can be used. Our recommendation for a successful NVC study using *in vivo* TPM is first to identify various vascular structures at low magnification, followed by high-magnification imaging to examine specific morphological features and cellular-level signaling. Most two-photon imaging platforms naturally incorporate a wide-field imaging setup. Therefore, we present a set of criteria (Table 2) for integrating these two imaging modalities to improve the accuracy and precision of vascular identification.

**Table 2:** Criteria to identify different vessel types by wide-field imaging, using either epifluorescence or natural light sources and two-photon microscopy.

	Wide-field imaging (5x objective)	Two-photon microscopy (25x objective, with vessel lumen dye)
**Pial arteriole**	(1) Clearly visualized on the brain surface.	(1) Above the pia mater.
	(2) Thick vessel wall.	(2) Diameter typically larger than 20 μm.
	(3) Marked vessel wall pulsatility.	(3) Thick vessel wall.
	(4) At the crossing point of an artery and a vein, the artery lies superficial to the vein.	(4)^a^ Long, dark, thin strips indicating fast movement of blood cells.
	(5) Direction of the flow in the arteries.	(5) Strong second harmonic generation (SHG)
	(6) Brighter color of the blood due to hemoglobin oxygenation.	
**Penetrating arteriole**	(1) Blurry but round cross-sectional shape at the initial diving segment into deep brain tissue.	(1) Round or elliptical shape of vessel cross-section at an x-y plane.
	(2) Thick vessel wall.	(2) Thick vessel wall.
	(3) Obvious vessel wall pulsatility.	(3) Dark thin strips indicating fast movement of blood cells.
		(4) Second harmonic generation (SHG)
**Capillary**	(1) Badly visualized, very blurry	(1) Dense and 3D network.
		(2) Typical diameter of 5-10 μm.
		(3)^b^ Dark, thick strips indicating slow movement of blood cells.
**Ascending venule**	(1) Blurry but round cross-sectional shape at the initial diving segment into deep brain tissue.	(1) Round or elliptical shape of vessel cross-section at the x-y plane.
	(2) Thin vessel wall.	(2) Thin vessel wall.
	(3) No obvious vessel wall pulsatility.	(3) Dark, thick strips indicating slow movement of blood cells.
**Pial venule**	(1) Clearly visualized on the brain surface.	(1) Above the pia mater.
	(2) Thin vessel wall.	(2) Diameter typically larger than 20 μm.
	(3) No obvious vessel wall pulsatility.	(3) Thin vessel wall.
	(4) At the crossing point of an artery and a vein, the vein lies beneath the artery.	(4) Short dark, thick strips, or individual dark cells indicate slow movement of blood cells.
	(5) Darker color of the blood due to hemoglobin deoxygenation.	

a The dark strips observed in TPM are generated by the laser scanning across blood cells, which are not labeled by the plasma dye and therefore appear as stripy shadows in TPM. The faster the blood flow, the less time each blood cell is captured during scanning, resulting in thinner strips, and vice versa.^63^ Furthermore, anesthetized mice exhibit slower blood flow compared with awake mice, and the expected strip thickness should be adjusted accordingly.

b Red blood cells passing through capillaries deform from their native biconcave shape into a parachute- or bullet-like form, with diameters matching the inner lumen of the capillaries. Under TPM, they appear as dark, thick strips.

## 4 Imaging Neurovascular Coupling

### 4.1 Neurovascular Coupling and Associated Cell Types

**Fig. 3:**
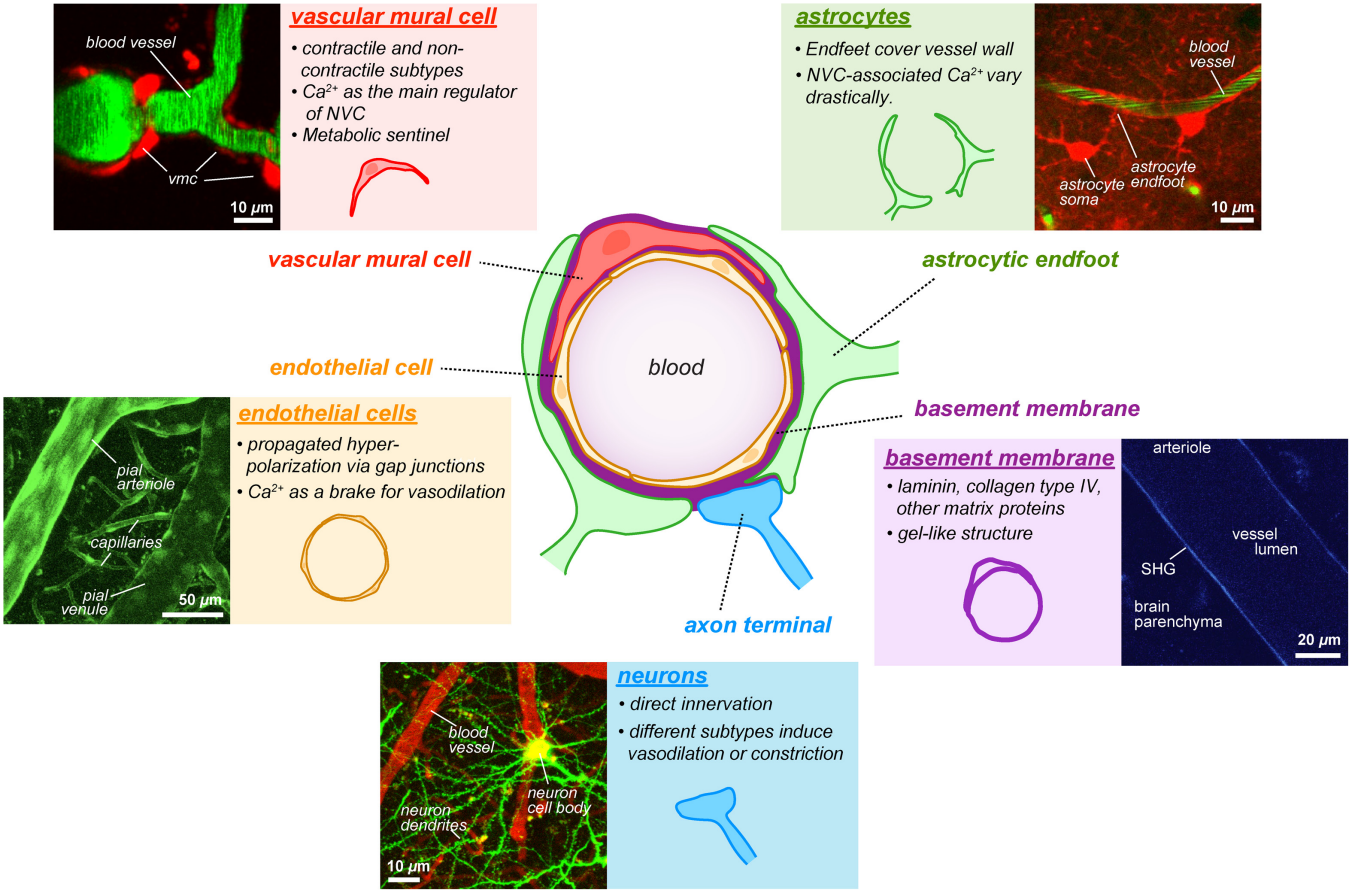
Principal cellular and non-cellular elements of the neurovascular unit, with their representative images from TPM *in vivo*. The panels show vascular mural cells signal imaged in NG2-DsRed transgenic animals, with intravenous (i.v.)-injected FITC-dextran to label plasma; astrocytes labeled by parenchymal injection of SR101, vessel lumen labeled with FITC-dextran; endothelium imaged in Tie2-GFP transgenic mice; neurons imaged in Thy-1 GFP mice, blood vessels labeled by i.v.-injected TRITC-dextran; and second-harmonic generation (SHG) signal delineating the vessel wall (basement membrane) in pial arteries and arterioles. Sources: Kucharz (*unpublished*), Cai et al. (*unpublished*).

NVC refers to the intricate physiological process by which neuronal activity drives local changes in CBF. This dynamic interaction relies on a network of signaling pathways involving various cells within the NVU, notably endothelial cells, vascular mural cells, astrocytes, and neurons (Fig. 3). The coordinated actions of these cell types ensure adequate nutrient and oxygen delivery to active neuronal regions, thereby sustaining cerebral homeostasis and function. Although most researchers believe NVC is vital for supplying metabolic needs for active neurons, it has been argued that baseline blood flow is sufficient for the elevated metabolic needs and suggested alternative functions of NVC, for example, temperature regulation and circulation of cerebrospinal fluid.^91^ Irrespective of these interesting considerations, vasodilation and increased blood flow occur during increased local neuronal activity. Overall, by unveiling the sophisticated signaling networks and functional interplay of endothelial cells, vascular mural cells, astrocytes, and neurons in NVC, these findings will depict a comprehensive picture of NVC and pave the way for novel therapeutic strategies targeting cerebrovascular dysfunctions associated with aging and neurodegenerative diseases.

#### 4.1.1 Endothelial cells

Endothelial cells form the innermost lining of the cerebrovascular system and are suggested to initiate and propagate vasodilatory signals. They express diverse ion channels and signaling pathways critical for this function. Recent studies have identified inward-rectifier potassium channels (Kir2.1) in endothelial cells as key sensors and amplifiers of neuronal activation signals.^92^^,^^93^ Increased extracellular $K^{+}$ during neuronal activation triggers endothelial hyperpolarization through Kir2.1 channels, propagating rapidly via gap junctions, and inducing relaxation of mural cells and thereby vasodilation. ATP-sensitive potassium ($K_{\mathrm{ATP}}$) channels, activated through adenosine signaling, further enhance endothelial hyperpolarization and vasodilatory responses.^94^ Endothelial intracellular calcium (${\mathrm{Ca}}^{2+}$) signaling, mediated by IP3 receptors (IP3Rs), transient receptor potential vanilloid type 4 (TRPV4), Piezo1, and transient receptor potential ankyrin 1 (TRPA1) channels, also significantly contributes to NVC.^9^^,^^95^^–^^97^ However, the precise role of endothelial ${\mathrm{Ca}}^{2+}$ in NVC remains unclear and somewhat controversial: The conventional view is that endothelial ${\mathrm{Ca}}^{2+}$ activates calcium-activated potassium channels (${\mathrm{IK}}_{\mathrm{Ca}}$ and ${\mathrm{SK}}_{\mathrm{Ca}}$), thereby promoting endothelial hyperpolarization.^98^^,^^99^ By contrast, a recent study reported that Piezo1-mediated ${\mathrm{Ca}}^{2+}$ rise in endothelial cells acts as a brake on hyperemia.^95^ In addition, at the arterial level, endothelial cells enriched in caveolae facilitate NVC independently of nitric oxide signaling,^100^ which effectively coordinate vascodilation.

#### 4.1.2 Vascular mural cells

Vascular mural cells, including SMCs located on arteries and arterioles, and pericytes on capillaries, play a critical role in regulating CBF. In the cerebral cortex, SMCs are ring-shaped. Pericytes display notable structural diversity and are categorized into ring-shaped, ensheathing, mesh, and thin-strand types.^62^^,^^101^ This classification reflects an organizational hierarchy, with pericytes progressively distributed along the vascular tree as it transitions from arteriolar side to venule side.^71^ It is widely acknowledged that SMCs, ring-shaped and ensheathing pericytes, contain alpha-smooth cell actin (αSMA) and are therefore contractile, whereas mesh and thin-strand pericytes contain low^102^ or no αSMA and are thus by most considered non-contractile.

SMCs, predominant around larger vessels, control blood flow via neuronal-induced vasodilation mediated by endothelial factors such as nitric oxide and prostaglandins.^2^^,^^48^^,^^103^^,^^104^ These factors cause SMC hyperpolarization, reducing calcium influx and leading to vessel relaxation. Pericytes, located along capillaries, actively modulate capillary diameter and blood flow.^73^ Neuronal activation induces local vasodilation in capillaries through astrocytic purinergic signaling and densely expressed pericyte ATP-sensitive potassium ($K_{\mathrm{ATP}}$) channel activation, which responds to metabolic signals.^105^ Furthermore, NVC elicited slowly propagated, localized vasodilation, which initiated at first- and second-order capillaries near penetrating arterioles, likely due to paracrine secretion of purinergic signaling from astrocytic end-feet.^47^ Precapillary sphincters, newly reported critical transition points between arterioles and capillaries, are characterized by their high α-smooth muscle actin expression and vascular lumen indentation.^43^ Precapillary sphincters and pericytes at first-order capillaries are highly sensitive regulatory zones, capable of significant and rapid alterations in capillary diameter and blood flow, thereby underscoring their essential role in cerebrovascular dynamics.^67^^,^^103^

#### 4.1.3 Astrocytes

Initial *in situ* studies indicated a significant role for astrocytes in NVC; however, early *in vivo* investigations yielded confusing results due to diverse astrocytic ${\mathrm{Ca}}^{2+}$ signaling patterns.^106^^–^^108^ Astrocytic ${\mathrm{Ca}}^{2+}$ activities range from widespread, prolonged events typically following vascular dilation,^82^^,^^109^^–^^111^ to smaller, rapid, localized events preceding vasodilation.^46^^,^^112^^,^^113^ These rapid signals are closely associated with neuronal activity and are thus highly relevant for NVC.^106^^,^^114^ Confusion increased further with studies using IP3R2 knockout mice, where astrocytic ${\mathrm{Ca}}^{2+}$ signaling is severely disrupted, yet NVC persists.^111^^,^^115^^,^^116^ However, recent investigations identified subtle but important alterations in NVC responses in these mice.^117^ Current understanding suggests astrocytic contributions to NVC differ across various vascular compartments: they significantly influence capillary dynamics, contribute to sustained dilation in penetrating arterioles, and regulate dilation transitions at precapillary sphincters between penetrating arterioles and capillaries.^82^^,^^104^^,^^118^ In addition, astrocytic activity and its contributions vary according to brain state, adding another layer of complexity.^119^^,^^120^ Thus, precise anatomical positioning and awake, *in vivo* experimental approaches are crucial for accurately elucidating the roles of astrocytes in NVC.

#### 4.1.4 Neurons

Neurons actively participate in NVC by directly influencing blood flow. Different neuronal subclasses release distinct vasodilators, each uniquely contributing to vascular responses.^121^ Increased excitatory neuronal activity elevates local oxygen and glucose demand, with recent findings highlighting direct glutamatergic innervation at arteriole-associated synapses.^122^ Conversely, inhibitory neuron activation introduces additional complexity, as various interneuron subtypes release diverse vasoactive substances.^123^^–^^129^ Notably, some inhibitory neurons induce vasoconstriction rather than vasodilation.^130^ Further complexity arises from interactions within neuronal networks, where excitatory neurons can activate inhibitory neurons.^131^ Thus, neuronal activation patterns can differentially recruit various neuronal populations depending on the specific mode of stimulation.^132^ The modus of stimulation is therefore a critical factor to consider when investigating specific neuronal subgroups in NVC.

#### 4.1.5 Non-cellular elements

The basement membrane is a thin layer of extracellular matrix located just outside the endothelial cells, providing structural support and a platform for cell–matrix interactions. Within this compartment, vascular mural cells are embedded, where they interact closely with the matrix to help maintain vascular stability and blood–brain barrier function.^133^^,^^134^ The core component of the basement membrane is fibrillar collagen and other non-centrosymmetric protein structures. Although the direct involvement of the basement membrane in NVC remains unclear, the basement membrane structural abnormalities accompany the vascular pathology in stroke and Alzheimer’s disease.^135^^,^^136^

### 4.2 Imaging Non-Cellular Elements During NVC

Labeling of the blood plasma with fluorescent dextrans remains the most widely used and reliable method for vascular lumen delineation in two-photon microscopy studies. High molecular weight dextrans prevent their rapid filtration by the kidneys, enabling imaging sessions that can last for several hours. This ensures high contrast for imaging vascular structures and helps avoid leakage, as demonstrated by the use of large, 2-MDa FITC-dextran, which does not extravasate, regardless of the postnatal time points.^137^ However, the use of high molecular weight dextrans is not without its limitations. Although generally stable, the presence of these tracers can alter the hemodynamic properties of the microvascular network, potentially slowing blood flow speeds in arterioles and venules, likely due to increased blood viscosity.^138^ In addition, >70-kDa dextrans are restricted to the capillary lumen’s central core rather than fully penetrating the glycocalyx,^139^ a negatively charged, ∼0.5-μm-thick layer lining the lumen side of endothelial cells in arterioles, capillaries, and venules. Although smaller molecular dyes, such as sodium fluorescein (∼0.376  kDa) and Alexa Fluor dyes (∼0.6 to 1.3 kDa), offer better glycocalyx penetration, their use comes with the increased risk of paracellular leakage, even in the healthy brain, which can also confound precise lumen measurements.^139^ Alternative approaches, such as fluorescent microspheres,^140^ or recently, genetically expressed albumin-based tracers exist,^141^ but their applicability may be limited by interactions with the endothelial glycocalyx and concerns about signal accuracy. The glycocalyx can both electrostatically repel microspheres or albumin and, regardless of the charge, limit their penetrance, acting as a molecular sieve, leading to poor access to the endothelial surface and an underestimation of vessel diameter.^142^^,^^143^ Overall, despite their drawbacks, fluorescent dextrans remain the most popular choice for accurate and reliable TPM-based vascular imaging *in vivo*. Complementary, SHG imaging can provide a label-free method for visualizing the collagen-rich extracellular matrix surrounding blood vessels. The strongest SHG signal is observed in arterioles, reflecting their thick basement membrane, whereas capillaries, venules, and veins, which lack substantial basal lamina, show little to no detectable signal^31^^,^^45^^,^^87^ (Table 3).

**Table 3:** Summary of key cellular components and fluorescent markers.

	Specific promoter for transgenic animals or viral vectors	Commonly used viral vector capsids	Chemical fluorescent dyes	Autofluorescence/SHG (indirect)
**Endothelial cells**	Tie2/Tek	(1) AAV-php.eB, AAV-php.V1 and AAV-php.N capsid injected in EC-specific Cre mice.	FITC-dextran*	Second-harmonic generation (SHG) to visualize arteries and arterioles.
	Cdh5			
			TRITC-dextran*	
			Texas Red-dextran*	
		(2) Bi30 capsid and BR1 capsid		
			Liver-secreted fluorescent blood plasma markers*	
			Lectin conjugates	
**Smooth muscle cells (vascular mural cells)**	ACTA2	AAV.PR capsid	/	/
	NG2			
	PDGFRβ			
**Pericytes (vascular mural cells)**	NG2	AAV.PR capsid	/	/
	PDGFRβ			
	KCNJ8			
	ABCC9			
**Astrocytes**	GFAP	(1) AAV5, AAV8 or AAV9	SR101	/
	GLAST	(2) AAV-php.eB, AAV-php.V1 and AAV-php.N capsid injected in astrocyte-specific Cre mice.	Rhod2	
	ALDH1L1		Fluo4	
	CX30		Oregon Green Bapta (Not astrocyte-specific)	
	FGFR3			
**Neurons**	Thy1	AAV-php.eB, AAV-php.V1 and AAV-php.N capsid injected in neuron-specific Cre mice.	Rhod2	/
	CamKII		Fluo4	
	Emx1		Oregon Green Bapta (Not neuron-specific)	
	DLX			
	GAD			
	hSyn			

Asterisks (*) denote indirect methods to visualize vessels by fluorescence in blood plasma.

### 4.3 Imaging Morphology and Signaling of Cells During NVC

**Fig. 4:**
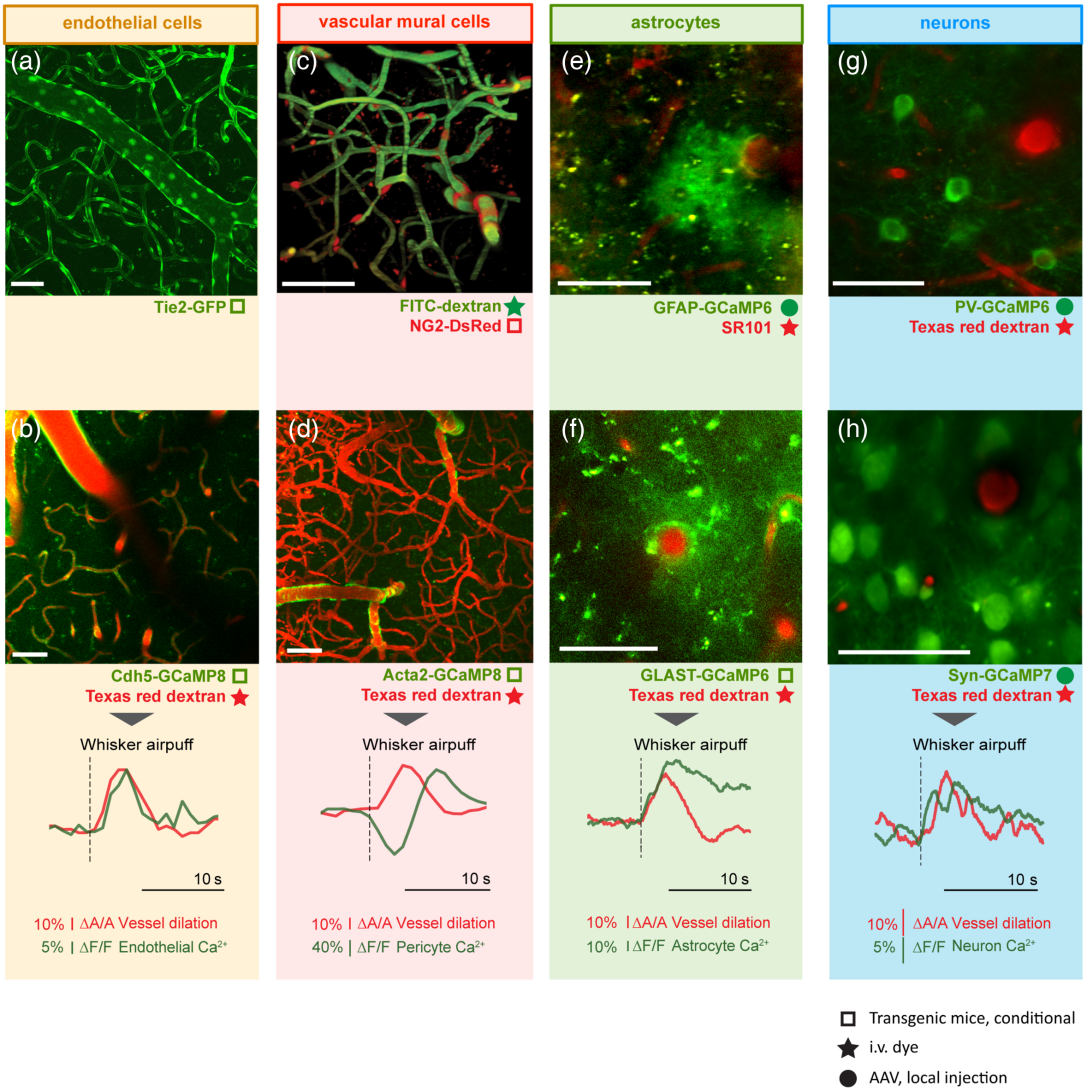
Representative images and traces of different neurovascular cells obtained by TPM. (a, b) TPM images and traces of vascular endothelial cells. Panel (a) was obtained from Tie2-GFP mice expressing GFP in endothelial cells. Panel (b) was obtained from Cdh5-GcaMP8 mice expressing green ${\mathrm{Ca}}^{2+}$ fluorescent indicator in endothelial cells and after intravenous injection of plasma dye Texas red-dextran. The representative traces were recorded during a whisker air puff in an awake mouse. (c, d) TPM images of vascular mural cells. Panel (c) was obtained from NG2-DsRed mice expressing DsRed in all vascular mural cells and after intravenous injection of plasma dye FITC-dextran. Panel (d) was obtained from ACTA2-GCaMP8 mice expressing green ${\mathrm{Ca}}^{2+}$ indicators in contractile mural cells, with intravenous injection of Texas red-dextran. The representative traces were recorded at a first-order capillary during a whisker air puff in an awake mouse. (e, f) TPM images of astrocytes. Panel (e) was obtained to study astrocyte ${\mathrm{Ca}}^{2+}$ by GCaMP6 indicator under the GFAP promoter in combination with intravenously introduced SR101 labeling of both astrocyte and vessel lumen. Panel (f) was obtained to study astrocyte ${\mathrm{Ca}}^{2+}$ by GCaMP6 indicator under the GLAST promoter in combination with Texas red-dextran-labeled blood vessel lumen. The representative traces were recorded during a whisker air puff in an awake mouse. (g, h) TPM images of neurons. Panel (g) was obtained to study inhibitory neuronal ${\mathrm{Ca}}^{2+}$ by GCaMP6 indicator under the DLX promoter to target PV interneurons specifically in combination with Texas Red-dextran-labeled blood vessel lumen. Panel (h) was obtained to study neuronal ${\mathrm{Ca}}^{2+}$ by GCaMP7 indicator under the syn promoter to target all neurons in cortical layer II/III in combination with Texas red-dextran-labeled blood vessel lumen. The representative traces were recorded during a whisker air puff in an awake mouse. The method used to introduce these dyes is indicated in the following way: “solid star”=i.v. dye; “solid circle”=AAV, locally injected; “open square”=transgene, conditional. Scale bar on all panels: 50  μm.

With the rapid development of cell-type–specific fluorescent markers expressed in transgenic animals or delivered via viral vectors, vascular researchers now possess a powerful toolkit to investigate the vascular cells with TPM *in vivo*. Although transgenetic mouse lines offer powerful, cell-type-specific gene expression, recent advances in viral vectors offer potent alternatives that are often more convenient and rapid, particularly when complex breeding poses a barrier. Using the Cre/Lox system in these genetic manipulations provides knock-in/knock-out capabilities highly relevant in vascular biology research. Certain viral capsids exhibit strong tropism for specific cell types, whereas others are capable of crossing the blood–brain barrier (BBB) after intravenous injection, enabling widespread transduction throughout the brain. It is important to note that only relatively small promoters can be packaged into adeno-associated viral (AAV) vectors to achieve cell-type–selective expression. An alternative strategy is to insert LoxP sites into AAV constructs and deliver them to cell-type–specific Cre mouse lines. In this section, we present an overview of vascular cell type-specific promoters and fluorescent labeling strategies, with examples to showcase studying morphology and signaling of endothelial cells, vascular mural cells, astrocytes, and neurons (Fig. 4 and Table 3).

#### 4.3.1 Imaging the endothelium *in vivo*

Several strategies facilitate investigations into the development and function of the cerebrovascular endothelium. Transgenic mice engineered to endogenously express GFP or other fluorophores under the control of endothelial-specific promoters, such as Tie2/Tek, enable direct visualization of endothelial morphology and vascular dynamics. For instance, the Tie2-GFP mouse cortex clearly demonstrates these features. A widely used approach in genetically targeting the endothelial cell studies is Cre-LoxP recombination technology. For example, Cdh5-CreERT2 mice can be crossed with Cre-dependent reporter lines encoding fluorescent proteins, e.g., calcium indicators, or other conditional knock-in/knock-out alleles. More recently, newly engineered viral vector capsids have enabled intravenous delivery for efficient infection of endothelial cells throughout the mouse brain. Systemic administration of viral vectors with capsids such as AAV-php.eB, AAV-php.V1, or AAV-php.N in Cre-dependent mouse lines allows selective expression of fluorescent cargos in endothelial cells.^144^^–^^146^ Alternatively, endothelial cell-specific viral vector capsids AAV-Bi30 and AAV-BR1 provide even more convenient and equally efficient expression in wild-type mice.^146^^,^^147^ In addition to direct genetic approaches, chemical fluorescent dyes are widely used for reliable, indirect labeling of brain endothelium (as discussed in the previous section).

Imaging endothelium is technically challenging as the structure of these cells is very thin, below the diffraction limit of TPM. Signal acquisition may also require long pixel dwell times during scanning, but is prone to motion artifacts. Alternatively, volumetric imaging of the local region using a piezo-motorized objective can improve the precision of signal acquisition and minimize out-of-focus artifacts,^51^ however, albeit at the cost of temporal resolution. Endothelial ${\mathrm{Ca}}^{2+}$ activity comprises both large, long-lasting events and small, short-duration flickers.^9^ Therefore, imaging settings should be carefully optimized to capture both types of signals.

Example 1: Usage of conditional knock-in transgenic Tie2-GFP mice (RRID:IMSR_JAX:003658) to visualize the EC-delineated vasculature [Fig. 4(a)]. The relevant usage examples can be found in the article.^31^

Example 2: Usage of conditional knock-in transgenic Cdh5-GCaMP8 mice (RRID:IMSR_JAX:033342) to study endothelial ${\mathrm{Ca}}^{2+}$ signals [Fig. 4(b)]. The vessel lumen is labeled with Texas red-dextran via intravenous injection. The representative traces show co-localized and co-varied vessel dilation and endothelial $Ca^{2+}$ signal upon whisker air puff in the awake state. Additional examples of this approach can be found in articles.^9^^,^^56^

#### 4.3.2 Imaging the vascular mural cells *in vivo*

As discussed in previous sections, vascular mural cells exhibit diverse morphologies, gene expression profiles, and contractile properties depending on their location within the cerebral vasculature. To study these cells, both universal and cell-type–specific promoters have been employed to target contractile SMCs, contractile pericytes, and non-contractile pericytes. NG2 and PDGFRβ are commonly used as universal promoters for labeling all mural cells, although they lack strict specificity. The NG2 promoter also targets NG2 glial cells,^148^ which are typically located away from blood vessels and can thus be distinguished from vascular mural cells based on spatial distribution. Similarly, the PDGFRβ promoter can drive expression in fibroblasts residing in the perivascular space, particularly around arteries and arterioles.^149^ This off-target expression necessitates caution when using PDGFRβ to study contractile mural cells.

To improve specificity, the ACTA2 promoter has been used to selectively target contractile mural cells,^150^ whereas the KCNJ8 and ABCC9 promoters are specific to non-contractile, $K_{\mathrm{ATP}}$ channel–rich pericytes.^151^ Transgenic mouse lines based on these promoters have enabled detailed investigations of the morphology and calcium signaling of distinct mural cell populations. Recent advances in AAVs offer promising new tools for pan-mural cell labeling in the mouse brain. For example, the AAV.PR capsid has shown potential for broad and efficient transduction of mural cells^152^ (although further studies are needed to evaluate its efficiency). By contrast, chemical fluorescent dyes currently lack the specificity and efficiency needed to label mural cells reliably.

Example 1: Usage of conditional knock-in transgenic NG2-DsRed mice (RRID:IMSR_JAX:008241) to visualize the morphology of vascular mural cells in an anesthetized state [Fig. 4(c)]. The vessel lumen is labeled with FITC-dextran via intravenous injection. The relevant usage examples can be found in the articles.^43^^,^^47^^,^^67^^,^^103^

Example 2: Usage of ACTA2-GcaMP8 conditional knock-in transgenic mice (RRID:IMSR_JAX:032887) to visualize the ${\mathrm{Ca}}^{2+}$ signals of contractile vascular mural cells [Fig. 4(d)]. The vessel lumen is labeled with Texas red-dextran via intravenous injection. The representative traces illustrate co-localized vessel diameter change and pericyte $Ca^{2+}$ signals at first-order capillary upon whisker air puff in the awake state. The vasodilation phase is concurrent with a decrease of pericyte ${\mathrm{Ca}}^{2+}$, and the vasoconstriction phase (return to baseline) is accompanied by an increase and subsequent overshoot of pericyte ${\mathrm{Ca}}^{2+}$. The relevant usage examples can be found in articles.^28^^,^^60^

#### 4.3.3 Imaging astrocytes *in vivo*

Several promoters specifically target astrocyte expression of fluorophores. The most common is the GFAP promoter [Fig. 4(e), Example 1], although it shows some low-level neuronal expression^153^ and labels cortical astrocytes sparsely.^82^ Alternatives include ALDH1L1,^154^ Cx-30,^155^ or Slc1a3 (GLAST) [Fig. 4(e), Example 2].^156^ Many of these promoters are available in transgenic mouse lines and have also been adapted for viral vector delivery,^154^ typically using astrocyte-tropic AAV serotypes 5, 8, or 9.^157^ When selecting astrocyte-specific promoters, it is crucial to consider brain-regional differences in expression and emerging astrocyte subtypes within the same brain area.^52^

Fluorophore expression in astrocytes highlights their structure, labeling soma, major processes, and vessel-ensheathing end-feet. Fine processes appear as a symmetric, cloud-like fluorescence around the soma.^106^ Despite the gap-junction connection in principle allowing cytosol connection between all astrocytes, individual astrocyte outlines are often distinguishable due to weak labeling of neighboring cells^82^ [Fig. 4(e), Example 1]. Most studies track intracellular $Ca^{2+}$ as a proxy for astrocyte activity, although tools exist for other molecules such as cAMP,^158^ chloride,^159^ and adenosine.^160^ $Ca^{2+}$ dynamics are best described, but are highly variable, ranging from localized, brief signals to prolonged, spreading ones.^106^ Although strongly labeled somata draw focus, key functions may lie in subtler and spatially contained activities. For this reason, many studies of astrocytic ${\mathrm{Ca}}^{2+}$ activities have been made using a membrane-tethered ${\mathrm{Ca}}^{2+}$ indicator, to enhance its sensitivity to membrane near events.^30^^,^^161^^,^^162^ The membrane-bound version was initially primarily delivered through AAVs under the constitutive active GFAP promoter. However, more recent studies have used transgenic mouse lines, e.g., containing ALDH1L1 astrocyte-specific promoter combined with a Cre-dependent expression of the fluorophore.^110^

Chemical fluorophores are also available for astrocyte labeling. Specificity can be achieved through surface loading, where dye is applied to the cortex and spreads through the astrocytic syncytium via their gap junctions.^163^^,^^164^ However, this method has limited depth (a few hundred μm) and requires leaving the craniotomy exposed for up to an hour. Alternatively, injecting the dye improves penetration but may reduce their specificity for astrocyte labeling.^46^ One of the common labeling approaches involves Sulforhodamine 101 (SR101),^46^^,^^165^ which mainly targets astrocytes but can also enter oligodendrocytes after prolonged exposure.^166^ A particular advantage of SR101 is that the dye may be administered intravenously or intraperitoneal, and cross the blood–brain barrier to label vessel-proximal astrocytes after about an hour in the bloodstream.^52^ This application (Example 1) supports labeling of astrocytes that does not require direct access to the brain and is compatible with awake imaging through a fully sealed chronic cranial window.^106^ However, SR101 usage may bear some risks as well, as some reported hyperexcitability of parenchymal cells after SR101 labeling.^167^

Examples 1: An example of virally introduced astrocyte GCaMP6 ${\mathrm{Ca}}^{2+}$ indicator expression under the GFAP promoter (AAV5-gfaABC1D-lck-GCaMP6f,^162^ Addgene catalog #52924-AAV5) in combination with intravenously introduced SR101 labeling of both astrocyte and vessel lumen [Fig. 4(f)]. The relevant usage examples can be found in the article.^120^

Example 2: A transgenic expression of GCaMP6 ${\mathrm{Ca}}^{2+}$ indicator in astrocytes by crossing a GLAST-Cre promoter mouse line (Slc1a3^tm1(cre/ERT2)Mgoe^, MGI:3830051)^168^ with a GCaMP6flox strain (B6J.Cg-*Gt(ROSA)26Sor*^*tm95.1(CAG-GCaMP*6*f)Hze*^/MwarJ, RRID: IMSR_JAX:028865), in combination with Texas red dextran-stained blood vessel lumen [Fig. 4(f)]. During a whisker air puff in an awake mouse, the representative traces show astrocyte ${\mathrm{Ca}}^{2+}$ signal along with diameter changes in the adjacent vessel. The relevant usage examples can be found in the article.^120^

#### 4.3.4 Imaging neurons *in vivo*

Neurons are the cell type most extensively studied using TPM, with a wide range of approaches developed to interrogate their function *in vivo*.^11^^,^^169^^,^^170^ Neurons have undergone extensive classification with regard to subgroups, differences in gene expression, and functionality between distinct brain regions.^171^^,^^172^ Nonetheless, it is often advantageous to use broadly active neuronal promoters, such as Syn or Emx1. More specific promoters can restrict expression to excitatory neurons (e.g., Thy1, CamKII) or GABAergic neurons (e.g., Dlx, GAD). These options are readily available either as transgenic mouse lines or via AAV-based delivery. Moreover, promoters can be combined with enhancers to refine targeting to specific neuronal subgroups.^173^ Thus, the choice of promoter should be guided by the experimental focus and desired cell-type specificity. Equally important is the consideration of neuronal response times, which occur on the order of tenths of a millisecond and therefore necessitate the use of indicators with sufficiently fast kinetics for studies of ${\mathrm{Ca}}^{2+}$ dynamics.^174^

Example 1: An example of a parvalbumin interneuron (PV)-specific expression of GCaMP6 ${\mathrm{Ca}}^{2+}$ indicator targeted with the PV-specific E2 enhancer-based expression (pAAV-S5E2-GCaMP6, Addgene catalog #135632-AAV1). The ${\mathrm{Ca}}^{2+}$ indicator is used in combination with Texas red-dextran-labeled blood vessel lumen [Fig. 4(g)]. The relevant usage examples can be found in the article.^175^

Examples 2: An example of ubiquitous neuron expression of the GCaMP7 ${\mathrm{Ca}}^{2+}$ indicator under the synapsin (Syn) promoter (AAV9-syn-jGCaMP7f, Addgene catalog #104488-AAV9) in combination with Texas red-dextran-labeled blood vessel lumen [Fig. 4(h)]. Upon whisker air puff to the awake mouse, the representative traces show co-localized vessel diameter changes and neuronal ${\mathrm{Ca}}^{2+}$ signal. The relevant usage examples can be found in the article.^120^

### 4.4 Choosing Between Anesthetized and Awake Animals for NVC Studies

When NVC is investigated *in vivo*, one of the principal decisions to make is deciding between experimenting on an anesthetized or awake animal. Several aspects should be considered when making this choice. The obvious one is the confounding impact of anesthetics. Most anesthetic agents substantially influence neurovascular dynamics by altering vascular tone and NVC mechanisms.^176^ Previous studies have demonstrated that anesthesia can suppress neuronal activity and modify vascular responses, potentially affecting experimental outcomes.^177^^–^^179^ Consequently, imaging conducted in awake, behaving animals may be considered preferable, as it provides a more accurate representation of genuine neurovascular interactions.^180^

**Fig. 5:**
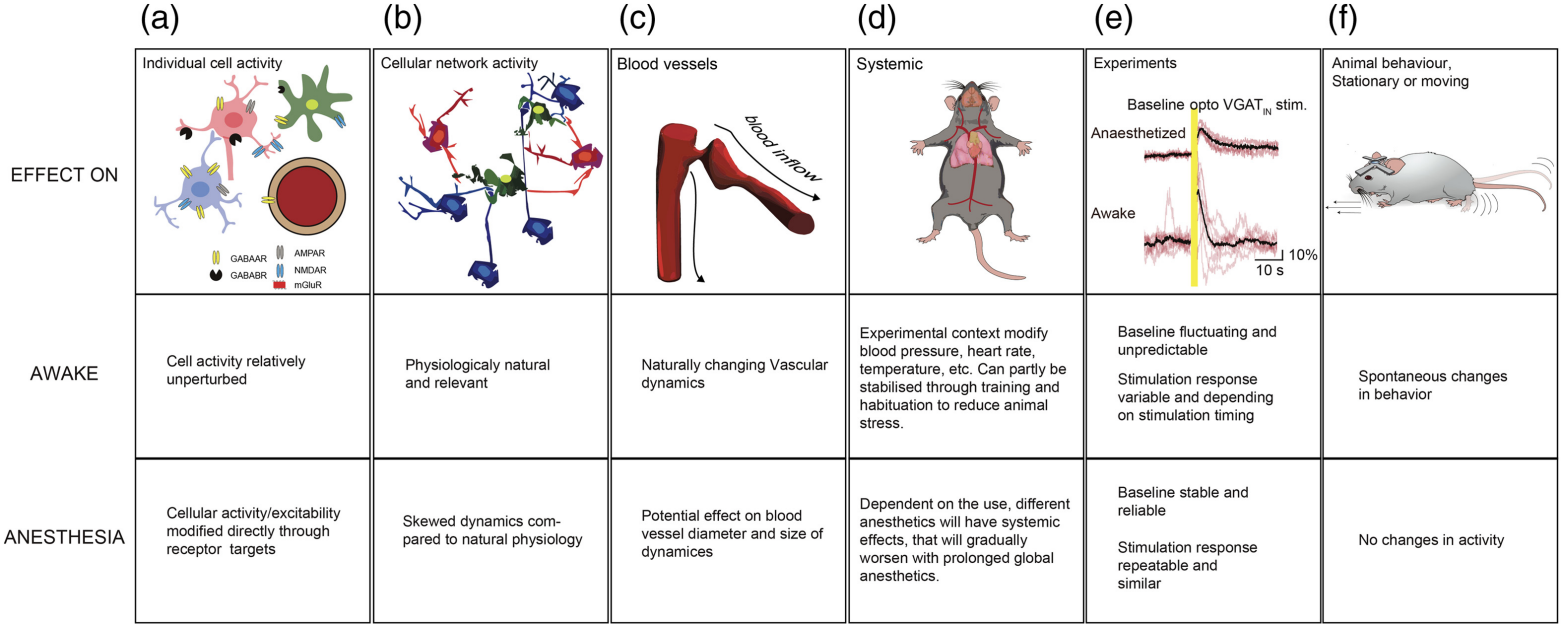
Pros and cons of conducting an *in vivo* neurovascular coupling study in awake versus anesthetized animals, with regard to aspects critical for the outcome of the investigation. The different aspects (a–f) are illustrated by drawings by the authors, except (e), which shows original PA dilation data from a study using optogenetic stimulation of interneurons *in vivo* in barrel cortex.^30^

Nevertheless, the decision regarding the use of anesthesia is complex, and experiments conducted in awake animals are not invariably superior (Fig. 5). The optimal approach depends strongly on the specific cellular mechanisms underlying NVC under investigation. Regardless of the anesthetic used, all compounds inevitably affect the cells involved in NVC regulation, with the extent of this influence varying according to the experimental focus. Investigations centered on the roles of specific neuronal subtypes^120^^,^^124^^–^^126^^,^^128^ or astrocytic contributions^30^^,^^106^^,^^110^^,^^181^ are particularly susceptible to significant anesthetic-induced alterations. This susceptibility arises because anesthetics act on receptors rather than specific cell types, so any cell expressing the anesthesia-targeted receptor can be affected [Fig. 5(a)].

In addition, anesthetics indirectly affect these cells by modifying neuronal network activity, which influences the function of the investigated cellular populations [Fig. 5(b)]. Common general anesthetics such as isoflurane, dexmedetomidine, and alpha-chloralose primarily target the GABA_A receptor,^182^ boosting inhibitory circuits, while ketamine functions as an NMDA receptor antagonist, silencing the glutamatergic excitatory pathways. The density and distribution of GABAergic and glutamatergic synapses vary significantly across neuronal populations and networks.^183^ Consequently, global anesthetic-induced inhibition does not uniformly suppress neuronal activity but disproportionately affects some neuronal connections more than others.^184^ This selective modulation leads to alterations in neuronal connectivity patterns that do not reflect a diminished version of the natural awake state, thus precluding direct comparisons to authentic NVC regulation. The fundamental disparities between anesthetized and awake brain states are highlighted by recent studies demonstrating differential dynamics of NVC events under these two conditions.^30^^,^^185^

The differences between NVC responses in awake versus anesthetized mice become apparent in studies examining vascular dilation events at the penetrating arteriole (PA) and capillary junction [Fig. 5(c)]. Hemodynamic responses induced by stimulation in anesthetized mice are typically stereotypical, consistently initiating at the capillary segment nearest the PA.^47^^,^^48^ Conversely, recent studies in awake mice have demonstrated highly heterogeneous patterns of dilation.^185^ This heterogeneity likely results from shifts in local regulatory mechanisms related to varying brain states and the integration of inputs from multiple brain regions.^186^ Despite such variability, NVC responses in awake mice appear to exhibit greater specificity toward naturally localized neuronal activations compared with anesthetized mice.^185^ Also, critically important are the central effects of the anesthetics [Fig. 5(d)], which may change breathing pattern, lower blood pressure, and heart rate, especially when not implemented correctly.^187^ These systemic effects of anesthesia may affect baseline measurements, skewing the neurovascular assessments.

Although these considerations strongly advocate for conducting NVC investigations in awake animals, they simultaneously highlight challenges arising from variability in vascular dynamics. Experimental protocols typically involve controlled stimulation aimed at inducing changes relative to a perceived baseline; however, establishing a reliable baseline is challenging in non-sedated animals [Fig. 5(e)]. Indeed, recent studies have observed negative hemodynamic responses resulting from interventions conducted while animals were asleep.^188^ Furthermore, awake animals frequently transition between spontaneous activities such as locomotion, blinking, or auditory responses, each inducing widespread vasodilation across multiple brain regions.^189^ Although behavioral paradigms^190^^,^^191^ and stringent inclusion criteria based on external indicators of brain state can partially mitigate these issues,^119^^,^^192^^,^^193^ establishing a consistent baseline remains difficult [Fig. 5(f)]. Consequently, if the primary focus of the NVC investigation is on mechanisms directly related to vascular contractility, the use of anesthesia may be advantageous.^43^ In short, the suppression of spontaneous dilations by general anesthetics provides a more stable baseline for experiments.

## 5 Challenges and Future Directions

### 5.1 Development of New Genetically Encoded Fluorescent Indicators

Recent advancements in genetically encoded fluorescent indicators have significantly expanded our capabilities in monitoring cellular and subcellular dynamics with high spatial and temporal precision using TPM. In particular, we highlight novel fluorescent indicators sensitive to voltage, potassium, and neuromodulators, which offer improved performance. Although they were initially developed for neuronal research, their intriguing applications in vascular research could potentially be revolutionary.

Voltage sensors have seen significant advancements, allowing for unprecedented visualization of cellular electrical activity in the brain. In particular, engineered voltage indicators such as the ArcLight,^194^^–^^196^ JEDIs,^197^ the ASAPs,^198^ and rhodopsin-based sensors^199^^,^^200^ offer faster, brighter, and more sensitive readouts, enabling reliable detection of rapid voltage fluctuations even in deep tissue layers of awake, behaving mice. Recent studies suggest that the propagation of hyperpolarization in endothelial and vascular mural cells acts as an “information highway,” transmitting hyperpolarizing signals and conveying metabolic demands from capillaries to upstream arterioles and arteries.^60^^,^^71^ Visualizing electrical signals in brain vascular cells and understanding how these signals are coordinated will mark a significant breakthrough and push the frontiers of neurovascular research.

Parallel developments have also emerged in potassium sensors, addressing the critical role of potassium ions in neuronal function. Multiple biosensors, including GINKOs and RGEPOs and nanosensors, have been developed to monitor intracellular and extracellular potassium in the brain.^201^^,^^202^ Further, through structure-guided design, KRaION1 and its engineered derivatives display improved sensitivity to potassium.^203^ As $K_{\mathrm{ir}}$ channels and $K_{\mathrm{ATP}}$ channels are suggested to play an important role in the initiation and propagation of NVC signals, and astrocytes are recognized as an important mediator of $K^{+}$ siphoning,^204^ investigation of potassium in the vascular cells will provide unprecedented insights into mechanisms in health and disease.

Finally, significant progress has been made in the development of sensors for neuromodulators and neuropeptides, including norepinephrine, serotonin, dopamine, histamine, and acetylcholine.^205^^–^^210^ In the awake brain, these signaling molecules play crucial roles in regulating basal vascular tone and NVC. However, the specific cell types involved and the underlying signaling pathways of NVC remain largely unclear. Widespread application of these advanced sensors will enable a more comprehensive understanding of how different brain regions communicate and coordinate, as well as how NVC is precisely regulated across time and space. This progress will also pave the way for deeper insights into vascular-associated pathological conditions.

Together, these state-of-the-art fluorescent indicators substantially expand the methodological toolbox available for neurovascular physiology, facilitating intricate exploration of physiological function and dysfunction through *in vivo* two-photon imaging techniques.

### 5.2 Advancements in New Imaging Techniques

Recent advances in new imaging technologies have significantly expanded our ability to investigate neural and vascular function with high spatial and temporal resolution. Several methods now offer powerful platforms to address pressing needs for *in vivo* vascular imaging, including the detection of rapid vascular signaling in 3D volumes and high-resolution measurements of vasculature deep within the brain.

(i) One-photon and two-photon light-sheet microscopy have emerged as an option for large-scale *in vivo* brain imaging. Unlike conventional point-scanning TPM, light-sheet approaches illuminate an entire plane orthogonal to the detection axis, enabling rapid volumetric acquisition with reduced photobleaching.^211^^–^^213^ However, not all light-sheet designs are compatible with in vivo mouse brain imaging. Conventional systems typically require two vertically oriented objectives, which limit accessibility to the mouse brain and are better suited for smaller animals such as zebrafish or fruit flies.^214^^,^^215^ To address this limitation, several single-objective designs have been developed that are suitable for *in vivo* rodent studies.^216^^,^^217^ Further innovations, such as structured illumination and near-infrared II (NIR-II) excitation, have improved both axial resolution and imaging depth.^218^^,^^219^
(ii) Bessel beam-based TPM enables high-speed volumetric imaging without the need for mechanical axial scanning.^220^ By generating an axially extended excitation focus, it transforms lateral scans into volumetric recordings. This is especially well-suited for capturing dynamic vascular events, such as vessel diameter changes and red blood cell flow, where temporal precision and volumetric information are essential.^221^ In awake mice, this technique has achieved volumetric imaging of blood flow at rates approaching 100 volumes per second.^222^ However, due to the extended excitation focus, a major drawback of this method is the reduction in spatial resolution, particularly along the z-axis.
(iii) Light field microscopy (LFM) provides a scan-free alternative for volumetric imaging by capturing both spatial and angular light information in a single camera exposure.^223^ Recent developments in Fourier and diffuser-based architectures have overcome previous limitations in spatial resolution and field of view. LFM is particularly well-suited for imaging complex, branching vasculature in 3D with high throughput and without mechanical scanning.^224^ Despite these advantages, LFM also has notable drawbacks, including lower image quality and limited penetration depth.^225^
(iv) Three-photon microscopy has become a powerful tool for deep-tissue vascular imaging.^226^ Using long-wavelength excitation (typically ∼1300 to 1700 nm) further minimizes scattering and absorption, allowing high-resolution visualization of vascular structures at depths exceeding 1 mm.^227^ This enables researchers to study vascular morphology, flow dynamics, and NVC in deep cortical and subcortical regions with minimal invasiveness.^228^^,^^229^ However, the high costs of the infrastructure, implementation, and running of three-photon microscopy setups are still a major limiting factor.

Collectively, these advanced imaging approaches highlight a shift toward fast, light-efficient, and computationally enhanced volumetric methods to offer new insights into the structure and function of the cerebral microvasculature.

### 5.3 Key Questions to be Addressed in NVC

Important knowledge gaps remain regarding neurovascular cells and NVC, many of which are well suited for investigation with TPM *in vivo*.

For endothelial cells, a major knowledge gap concerns the molecular mechanisms and signaling pathways underlying their role in NVC across different vascular segments. Our understanding is still at an early stage, particularly regarding how endothelial ${\mathrm{Ca}}^{2+}$ dynamics and hyperpolarization coordinate the initiation and propagation of vasodilatory signals. In addition, endothelial cells at arterioles are contacted by neuronal projections,^122^ whereas those at capillaries are tightly ensheathed by astrocytic end-feet. The precise molecular signals transmitted from neurons and astrocytes to regulate endothelial function remain poorly defined and represent the active research focus of neuroscientists using TPM.

For vascular mural cells, they are connected with EC via gap junctions and other possible paracrine signaling pathways.^9^^,^^230^ However, the exact signaling mechanisms and the physiological functions by which pericytes communicate with astrocytes and interact with endothelial cells remain poorly characterized. A significant effort is directed toward understanding how pericyte dysfunction contributes to persistent cerebral hypoperfusion and blood–brain barrier leakage observed in pathologies such as stroke and neurodegenerative diseases and how to mitigate those pathologies.

For astrocytes, despite evidence that astrocytic ${\mathrm{Ca}}^{2+}$ elevations trigger both vasodilation and vasoconstriction, significant uncertainties exist regarding the precise physiological conditions and factors that dictate the switch between these opposing vascular responses.^231^^,^^232^ Further research may provide answers to how astrocytic signaling integrates with neuronal, mural cells, and endothelial signals to produce coordinated vascular responses across diverse brain regions and activity states.

By revealing the complex signaling networks and functional interactions among endothelial cells, vascular mural cells, and astrocytes in NVC, TPM provides a powerful tool to advance these discoveries, ultimately paving the way for new therapeutic strategies against cerebrovascular dysfunctions linked to aging and neurodegenerative diseases.

## Data Availability

Data sharing is not applicable to this article, as no new data was created or analyzed.
